# A novel comparative pattern analysis approach identifies chronic alcohol mediated dysregulation of transcriptomic dynamics during liver regeneration

**DOI:** 10.1186/s12864-016-2492-x

**Published:** 2016-03-25

**Authors:** Lakshmi Kuttippurathu, Egle Juskeviciute, Rachael P Dippold, Jan B. Hoek, Rajanikanth Vadigepalli

**Affiliations:** Daniel Baugh Institute for Functional Genomics and Computational Biology, Department of Pathology, Anatomy and Cell Biology, Thomas Jefferson University, Philadelphia, PA 19107 USA; MitoCare Center for Mitochondrial Research, Department of Pathology, Anatomy and Cell Biology, Thomas Jefferson University, Philadelphia, PA 19107 USA

## Abstract

**Background:**

Liver regeneration is inhibited by chronic ethanol consumption and this impaired repair response may contribute to the risk for alcoholic liver disease. We developed and applied a novel data analysis approach to assess the effect of chronic ethanol intake in the mechanisms responsible for liver regeneration. We performed a time series transcriptomic profiling study of the regeneration response after 2/3^rd^ partial hepatectomy (PHx) in ethanol-fed and isocaloric control rats.

**Results:**

We developed a novel data analysis approach focusing on comparative pattern counts (COMPACT) to exhaustively identify the dominant and subtle differential expression patterns. Approximately 6500 genes were differentially regulated in Ethanol or Control groups within 24 h after PHx. Adaptation to chronic ethanol intake significantly altered the immediate early gene expression patterns and nearly completely abrogated the cell cycle induction in hepatocytes post PHx. The patterns highlighted by COMPACT analysis contained several non-parenchymal cell specific markers indicating their aberrant transcriptional response as a novel mechanism through which chronic ethanol intake deregulates the integrated liver tissue response.

**Conclusions:**

Our novel comparative pattern analysis revealed new insights into ethanol-mediated molecular changes in non-parenchymal liver cells as a possible contribution to the defective liver regeneration phenotype. The results revealed for the first time an ethanol-induced shift of hepatic stellate cells from a pro-regenerative phenotype to that of an anti-regenerative state after PHx. Our results can form the basis for novel interventions targeting the non-parenchymal cells in normalizing the dysfunctional repair response process in alcoholic liver disease. Our approach is illustrated online at http://compact.jefferson.edu.

**Electronic supplementary material:**

The online version of this article (doi:10.1186/s12864-016-2492-x) contains supplementary material, which is available to authorized users.

## Background

Multi time-series microarray measurements examining the temporal variation in gene expression across time points are useful in exploring the molecular mechanisms controlling biological processes. A variety of statistical techniques and methods are available to analyze time series transcriptomic data sets. Traditional statistical methods utilized significance tests, clustering methods and regression models to find differentially regulated genes. However, the dynamic nature of the effects of experimental perturbations makes it difficult to explore beyond the dominant aspect of the data. Conventional analysis approaches such as Linear Models for Microarray Data (LIMMA) [1], significance analysis of microarray (SMA) [2], Analysis of variance (ANOVA) [3] are based on statistical significance tests to identify differentially regulated genes. These are followed by clustering methods to classify gene expression profiles into groups of similar co-expression patterns. For instance, Short Time-series Expression Miner (STEM) tool [4] and Weighted Gene Correlation Network Analysis (WGCNA) [5] utilize a clustering based approach to identify temporal patterns from gene expression data. In contrast, regression modeling [6–8] is used to bridge the gap between the expression changes and a particular phenotype. However, these methods focus on retrieving the dominant gene expression profiles obtained from a direct comparison between the normal versus disease data sets. In our study, we introduce a comparative dynamic pattern analysis that can be a novel alternative with the potential to uncover aspects that are masked or hidden in conventional analyses. We applied this approach to a data set from rat liver to investigate the effect of chronic ethanol intake on the regeneration process following partial hepatectomy (PHx).

The liver has a remarkable ability to regenerate after injury. PHx is a widely used animal model to study the progression of regeneration, in which left lateral and medial lobes are surgically removed [9–15]. Multiple factors and signaling pathways work synergistically to achieve the goal of successful liver regeneration. From the quiescent G0 state, hepatocytes enter G1, the pre-replicative phase within 6 h post PHx. This is followed by hepatocyte and non-parenchymal cell replication and G2-M phases (12–96 h). This progression is continued until the termination phase (96–168 h) when the liver restores its original tissue mass [16, 17]. PHx induces activation of stress signals and hemodynamic changes mediated by adrenergic and purinergic agonists that drive liver cells from G0 phase to enter the cell cycle and induce proliferation [9, 18, 19]. Various factors important for the regeneration process are activated in the immediate early phase, prior to cell cycle entry [11, 20, 21]. These include signals from cytokines, growth factors and proteases such as matrix metalloprotease-9 (MMP-9) [17, 22]. During this priming phase, Kupffer cells release cytokines triggering a pro-inflammatory response, following which hepatocytes enter the replicative phase, followed by cytokine production of other non-parenchymal cells such as hepatic stellate cells, cholangiocytes and sinusoidal endothelial cells [9, 12, 23]. A better understanding of the regenerative process has potential clinical relevance in tumor resection and liver transplantation. Several experimental and computational studies have been conducted to investigate the dynamic changes in liver of multiple factors during liver regeneration [11, 13, 16, 17, 24–28]. However, despite its clinical importance, the underlying molecular mechanisms that drive the damaged liver to help restore its complex architecture are not completely understood.

Several external factors can cause damage to the liver and can limit the speed and efficiency of regeneration by disrupting the signals. Chronic ethanol consumption is one such factor; metabolism of ethanol leads to biochemical changes that affect the healthy functioning of the liver. This can develop into alcoholic liver disease (ALD), one of the primary causes of liver-related mortality [29–31]. Studies examining the effect of ethanol intake on liver function showed that chronic ethanol intake has the potential to impair hepatocyte replication by affecting the G1/S and G2/M transitions of the cell cycle [32] and can delay the process of regeneration post PHx [33–41].

DNA microarray technology has been used to identify global gene expression in the ethanol-adapted liver for different species [42–45]. A transcriptomic analysis of rat liver revealed that chronic ethanol consumption regulated mainly the genes implicated in the processes of signal transduction, transcription, immune response, and amino acid metabolism [45]. To our knowledge, a global level study of the gene expression mediated regulatory changes that take place in the liver during regeneration due to chronic ethanol intake has not been undertaken. Using a time series analysis of the early response phase (0-24 h) in the rat, we detected the differentially regulated genes in the chronic ethanol diet group compared to pair-fed controls during the initial phase of regeneration.

In the present study, we introduce a novel approach to the analysis of microarray data, and applied it to the analyzing the dynamic adaptation of the liver gene expression to chronic ethanol intake. We investigated the effect of ethanol intake on global gene expression at two levels: (i) corresponding to chronic intake, and (ii) when acutely perturbed from this adapted state through liver regeneration induced by 2/3^rd^ PHx. Conventional clustering approaches were informative as a first order analysis at the broad systems level, but fine-grained results on ethanol effects were eluding the typical cluster analyses due to the complexity of interpreting the results from numerous comparisons. To overcome this hurdle, we present a novel approach based on analysis of discretized expression patterns. Our approach compares the patterns of temporal expression progression across dietary groups, and represents the results using a pattern count matrix and associated circular visualization, enabling straightforward and intuitive identification of similarities and differences in the expression programs due to chronic ethanol intake. This approach permits us to focus on the results in a hierarchical manner by masking patterns with larger number of genes, revealing less obvious patterns with fewer gene members. Our novel comparative pattern analysis revealed new insights into ethanol-mediated molecular changes in non-parenchymal liver cells as underlying the defective liver regeneration phenotype.

## Results

### A novel approach for transcriptomic analysis by comparing dynamic gene expression response patterns across conditions

**Fig. 1:**
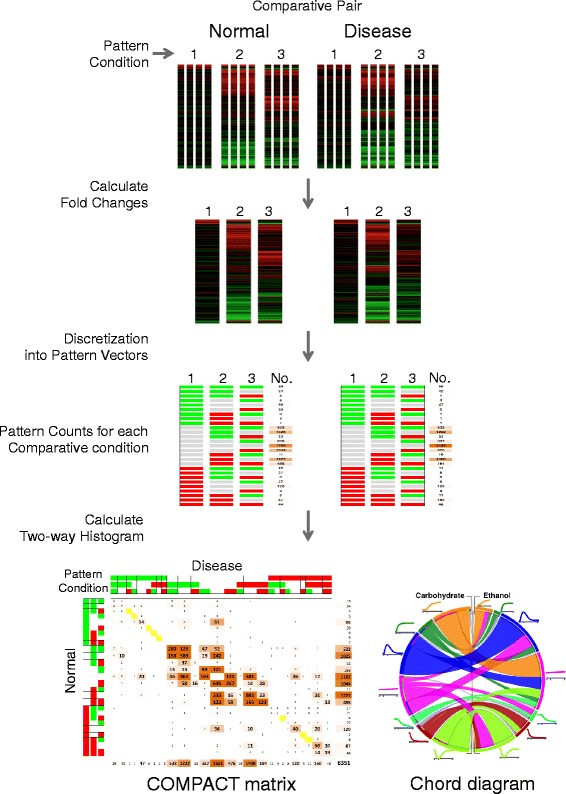
A schematic representation of the comparative pattern count (COMPACT) analysis for multifactorial transcriptomic data. The approach considers an experimental design in which the transcriptomic effects of two conditions, termed “Comparative Pair”, (e.g., Disease versus Normal) are evaluated at multiple levels of another factor, termed “Pattern Set”, (e.g., time points, drug dose, weight group, cell type, etc.). The following explanation considers Disease versus Normal comparison at multiple time points, for illustration purposes. First, the differential gene expression at each time point is computed relative to appropriate, likely time point specific, control conditions. The time series data is averaged across replicates within each sample group, and discretized into up-, down-, and no-regulation based on a threshold of differential expression level. The sample groups are divided into two sets based on the comparative pair: Disease versus Normal. Within each set, the discretized time series expression data is collated for each gene into a pattern vector. The number of genes corresponding to each of the patterns within Disease or Normal sets provides a univariate distribution of patterns. The intersection between the Disease versus Normal pattern count distributions (i.e., the two-way histogram) exhaustively considers all possible comparative patterns, yielding a COMPACT matrix. The elements of the COMPACT matrix are based on pair-wise gene counts of the corresponding patterns, i.e., for the comparative pair Disease and Normal, the element at the i^th^ row and j^th^ column of the matrix contains the number of genes that show an i^th^ expression pattern in Normal and j^th^ expression pattern in Disease. The diagonal of the matrix represents those genes showing a common response and the off-diagonal elements represent the genes showing an altered temporal response between the two comparative conditions. The organization of the COMPACT matrix lends itself to a layered exploration of the genes with common, novel and altered differential expression patterns between the conditions in the Comparative Pair, as illustrated in the analysis below (e.g., Figs. 6 and 7). The COMPACT matrix, or subsets thereof, can be visualized using a chord diagram, providing additional means for layered exploration of the data, particularly when the matrix is relatively less sparse (e.g., as illustrated in Fig. 8)

We developed a novel bioinformatics approach focusing on comparative pattern counts (COMPACT) to enable a hierarchical exploration, analysis and visualization of multi-dimensional transcriptomic data (Fig. 1). This COMPACT approach was particularly suited to highlight expression patterns that are typically “crowded out” in conventional analyses. The approach considers an experimental design in which the transcriptomic effects of two conditions, termed “Comparative Pair”, (e.g., Disease versus Normal) are evaluated at multiple levels of another factor, termed “Pattern Set”, (e.g., time points, drug dose, weight group, cell type, etc.). The following explanation considers Disease versus Normal comparison at multiple time points, for illustration purposes. First, the differential gene expression at each time point is computed relative to appropriate, likely time point specific, control conditions. The time series data is averaged across replicates within each sample group, and discretized into up-, down-, and no-regulation based on a threshold of differential expression level. The sample groups are divided into two sets based on the comparative pair: Disease versus Normal. Within each set, the discretized time series expression data is collated for each gene into a pattern vector. The number of genes corresponding to each of the patterns within Disease or Normal sets provides a univariate distribution of patterns. The intersection between the Diseases versus Normal pattern count distributions (i.e., the two-way histogram) exhaustively considers all possible comparative patterns, yielding a COMPACT matrix. The elements of the COMPACT matrix are based on pair-wise gene counts of the corresponding patterns, i.e., for the comparative pair Disease and Normal, the element at the i^th^ row and j^th^ column of the matrix contains the number of genes that show an i^th^ expression pattern in Normal and j^th^ expression pattern in Disease. The diagonal of the matrix represents those genes showing a common response and the off-diagonal elements represent the genes showing an altered temporal response between the two comparative conditions. The patterns are arranged along rows and columns such that the null pattern representing “No differential expression” broadly separates the up- and downregulation at the first time point. This approach allows us to take an overall view for systems level differences in expression patterns between the Diseases versus Normal groups and then systematically explore the gene regulatory patterns that are altered in specific ways. The organization of the COMPACT matrix lends itself to a layered exploration of the genes with common, novel and altered differential expression patterns between the conditions in the Comparative Pair, as illustrated in the analysis below. The COMPACT matrix, or subsets thereof, can also be visualized using a chord diagram, providing additional means for layered exploration of the data, particularly when the matrix is relatively less sparse. Our approach is illustrated online at http://compact.jefferson.edu

We utilized the novel COMPACT approach to analyze and visualize changes in differential regulation of genes in the rat liver during response to PHx, in control and chronically ethanol-fed animals. We explored patterns in the time-course of gene expression profiles; the major findings are explained in the following sections.

### Liver transcriptome shows nearly complete adaptation to chronic alcohol consumption

**Fig. 2:**
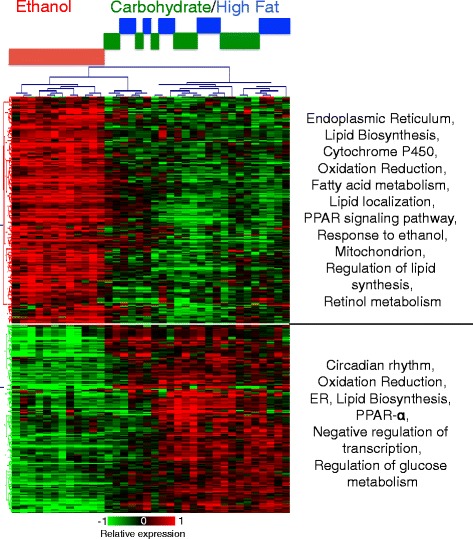
Differential gene expression in the liver at the baseline state across the three dietary groups. The heat map includes 400 up-and down-regulated genes in ethanol-adapted condition compared to the isocaloric Carbohydrate and High Fat diet samples. Ethanol group samples cluster separately from the Carbohydrate and High Fat group samples, with Carbohydrate and High Fat group samples showing no obvious sub-grouping. The statistically enriched processes, molecular functions and cellular localizations are shown next to the up-and downregulated gene sets

Our initial focus was to identify the persistent gene expression changes and associated cellular functions that are likely to be perturbed due to chronic ethanol feeding. We considered two isocaloric controls where the dietary calories derived from ethanol were replaced by carbohydrate or fat, respectively (Carbohydrate and High Fat dietary groups). Analysis of the gene expression data obtained from the liver samples (12 replicates per diet group, 3 dietary groups) revealed a total of 400 differentially regulated transcript clusters (334 annotated by RefSeq ID) in ethanol-adapted animals compared to either control diet (Fig. 2; Additional file 1: Table S1). There were 220 upregulated and 180 downregulated transcript clusters. Remarkably, nearly all of the average gene expression changes were well below 2-fold, with a wider range of expression across biological replicates. There was no evidence that certain samples were more likely to be on the higher or lower ends of the response range over many genes. The ethanol group showed distinct expression profiles and grouped separately from those of the isocaloric Control groups (Fig. 2). By contrast, the majority of the isocaloric Carbohydrate and High Fat control diet samples grouped together. However, within the two large sample clusters, one of the sub-clusters with control samples was in the same larger cluster as the ethanol group. These apparent ‘outlier’ control samples showed gene expression profiles that were intermediate between the ethanol group and other controls, with a subset of genes showing a differential expression trend that is similar to the ethanol group. There were 36 transcripts with significant differential gene expression in the isocaloric high fat samples relative to the isocaloric Carbohydrate samples, at average fold change > =1.5 and *q*-value < =0.2, i.e., there were relatively few genes that were differentially expressed between the two isocaloric control groups, supporting the interpretation that the gene expression in the two control groups is nearly indistinguishable. A total of 17 transcripts in the ethanol-responsive set of 400 also showed a response in the isocaloric high fat diet group. Eight genes in this set showed an opposite response between the Ethanol and isocaloric High Fat diet groups. Pathway analysis of differentially regulated genes indicated significant alterations in gene sets related to circadian rhythm, lipid and steroid metabolism, endoplasmic reticulum (ER) membrane-related functions, and mitochondrial functions, consistent with observed phenotypes of fatty liver and a shift in circadian rhythm in alcohol-adapted animals [46, 47]. These results indicate that the liver gene expression shows an adaptive response to chronic ethanol intake with relatively modest changes in average steady state expression levels.

### Partial hepatectomy induces broad changes in the liver transcriptome

We investigated whether acute perturbation such as liver regeneration induced by 2/3^rd^ PHx would elicit differential transcriptomic response across the three dietary groups. Our analysis of the gene expression time series data revealed a total of 6893 genes to be responsive to liver regeneration at one or more time points post PHx, in at least one of the three dietary groups. Remarkably, all three dietary groups showed broad transcriptomic changes independent of the regenerative outcome, implying that the regeneration deficiency observed in the Ethanol group may not be due to lack of differential gene expression broadly but due to an inappropriate transcriptional response along particular pathways/functions.

**Fig. 3:**
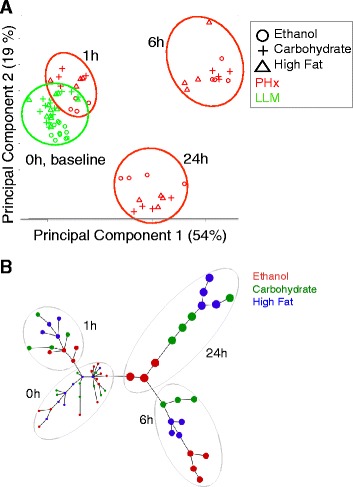
Ethanol-adapted liver shows distinct transcriptomic response to PHx. **a** Principal Component Analysis revealed sample separation across the first two components that captured most variability in the data. The sample grouped largely based on time points following PHx, with the Ethanol group separated from the control groups to a lesser degree. The 6 h PHx samples group separately from the 24 h samples indicating significant differences in gene expression over time. **b** A minimum spanning tree demonstrating the distinct clustering of the Ethanol group relative to the Carbohydrate and High Fat groups. The 1 h and 24 Ethanol group samples were located closer to the 0 h samples, relative to the Control groups at these time points, indicating deficiencies in mounting a response to PHx in the Ethanol group. The 6 h Ethanol group samples were furthest relative to the control groups indicating a potential larger scale of transcriptomic response at this time in the Ethanol group

We first sought to compare whether the dietary condition or PHx-induced temporal changes dominate the system response. We employed the widely used Principal Component Analysis (PCA) to identify putative sample clusters in a data-driven approach. PCA permits a visual and quantitative approach to investigating the similarities and differences between the overall gene expression responses across biological samples, and is particularly suited for uncovering patterns in high dimensional data. Our results reveal that the sample groups separate along the first two principal components based on temporal progression during regeneration (Fig. 3a). The magnitude of gene expression differences between the dietary groups appears to be less than that of time post PHx. Ethanol samples did not clearly separate from the other dietary groups within the first two PCs. However, the results demonstrated that the Ethanol group is most distinct from the Control group as shown by the qualitative separation between the diet groups at the baseline (excised liver samples at 0 h) and at 1 h and 24 h post PHx. The two control dietary groups clustered together at these time points. The 24 h samples were located in-between the 1 h and 6 h samples, as a large number of genes showed a transient differential expression only at 6 h and not at 1 h or 24 h. This interpretation is supported by the subsequent analysis of the gene counts corresponding to dynamic patterns as below. We further analyzed the differential gene expression response using a minimum spanning tree approach (Fig. 3b). This approach improves significantly over the very commonly used hierarchically clustering and is readily interpretable for cellular phenotypes and subtypes, and was used by us previously to organize trajectories of developmental phenotypes altered by ethanol [48]. Consistent with the PCA results, the minimum spanning tree structure was largely oriented based on the time points post PHx, and the dietary groups were distributed in distinct ways at each time point. The Ethanol group was closer to 0 h baseline samples at 1 h and at 24 h, indicating deficiencies in mounting a gene regulatory response to PHx. However, the Ethanol group at 6 h was the furthest of all the 6 h samples from the 0 h baseline group, suggesting a more pronounced differential gene expression at 6 h in the Ethanol group.

### Dynamic response pattern analysis revealed opposite regulation of proliferative and anti-proliferative processes in the control of liver regeneration

**Fig. 4:**
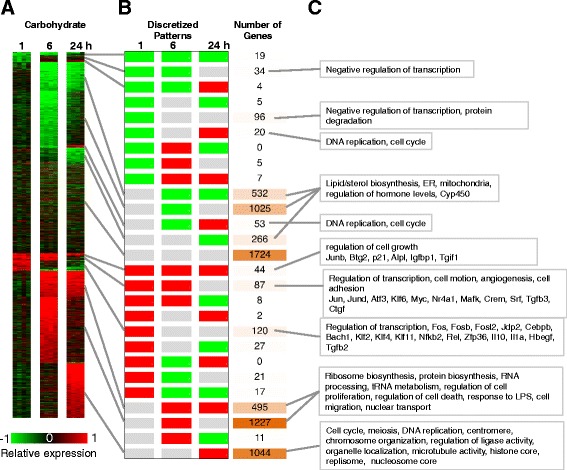
Differential gene expression patterns in response to PHx in the Carbohydrate group. **a** Average differential expression was computed at each time point post PHx, based on comparing PHx samples and the corresponding excised LLM tissue at *t* = 0 from the same animals. The genes are ordered based on the expression patterns shown in B. **b** A total of 27 discretized expression patterns based on three possible levels of regulation (up, down and no change) at each of the three time points (1, 6 and 24 h post PHx). The pattern counts are based on a differential regulation threshold of 1.75 fold. The majority of the genes were in the patterns that showed differential regulation at 24 h post PHx. A large set of genes showed no change in the Carbohydrate samples, but were included here, as these genes show differential regulation in other dietary groups. **c** A selection of statistically enriched processes and key genes in the discretized patterns

In order to better understand the effect of dietary treatment on gene expression during liver regeneration, we further analyzed the data for differences in the temporal patterns of differential gene expression response between the dietary groups, using a dynamic response pattern analysis (Methods) to classify the differential gene expression in the Carbohydrate control dietary group along 27 possible patterns (Fig. 4a). Investigation of statistically representative cellular functions and processes in these gene expression clusters yielded insights into the ordered progression through liver regeneration. The dominant patterns with large number of genes corresponded to differential expression at 6 h or 24 h time point post PHx (Fig. 4b). In contrast, there were relatively few genes (516 out of 6893) that showed an immediate early response at 1 h. This distribution is in agreement with the previous studies by our group and others that showed a significant surge in gene expression response in the 6 h time frame [24, 27, 49]. The immediate early liver regeneration response at 1 h post PHx in the Carbohydrate control animals contained patterns with relatively fewer genes than at later time points. A majority of these gene expression changes were transient at 1 h (120 up regulated, 96 down regulated), with a smaller fraction of genes showing a sustained response until 6 h (87 up regulated, 34 down regulated) or until 24 h (44 up regulated, 19 down regulated). The up regulated gene expression clusters were enriched for a number of transcription factors and regulators, as well as signaling factors. For example, the transcriptional regulators *Fos*, *Fosb*, *Fosl2*, *Jdp2*, *Bach1*, *Klf2*, *Klf4*, *Klf11*, *Nfkb2*, *Rel*, *Zfp36*, and the signaling factors *Il10*, *Hbegf*, *Tgfb2*, and *Gdf15* were transiently up regulated at 1 h.

Other transcriptional regulators such as *Jun*, *Jund*, *Atf3*, *Klf6*, *Myc*, *Nr4a1*, *Crem*, *Srf*, and factors *Tgfb3*, *Ctgf*, showed a sustained up regulation until 6 h. A smaller set of regulators such as *Junb*, *Btg2*, *p21*, *Alpl*, *Igfbp1*, and *Tgif1* showed a sustained up regulation until the 24 h time point. Several of these genes have been described to be critical to the initiation and progression of liver regeneration [9, 50–52]. The 1 h down regulated gene expression patterns were enriched for genes with functional annotation as negative regulation of transcription and protein degradation (Fig. 4c). These results indicate that the liver response not only includes activation of proliferative processes, but also contains an inhibition of the negative regulators of these functions, indicating an efficient tuning of the regenerative process.

Within the dominant expression patterns, a large majority of genes showed a transient up regulation (1227 genes) or down regulation (1025 genes) at the 6 h time point, while a smaller set of genes showed a sustained up or down regulation response from 6 h to 24 h (495 up and 532 down) (Fig. 4b). The sets of genes with transient or sustained up regulation in the 6 h to 24 h time frame were enriched for various processes critically supporting cell proliferation, including ribosome biosynthesis, protein biosynthesis, RNA processing, tRNA metabolism, cell migration, regulation of cell death, response to lipopolysaccharide (LPS), etc. (Fig. 4c). The clusters with down regulated genes at 6 or 24 h enriched for genes participating in lipid biosynthesis, regulation of hormone levels, and for several *Cyp450* family members.

Another dominant pattern was transcriptional up regulation by 24 h (1044 genes) (Fig. 4b). This cluster was enriched for genes participating in cell cycle, meiosis, DNA replication, chromosome organization, regulation of ligase activity, organelle localization, and microtubule activity. Correspondingly, the components of centromere, histone core, nucleosome core and replisome were over-represented in this set (Fig. 4c). A smaller set of genes also showed activation by 24 h but downregulation at 6 h (53 genes) or at 1 h (20 genes). These clusters were enriched for DNA replication and cell cycle genes. The 24 h activation results indicate a progression through the DNA replication phase of the cell cycle in this time frame, inline with the expected physiological dynamics of rat liver regeneration.

### Novel comparative pattern analysis revealed dominant and subtle differences in gene expression dynamics

**Fig. 5:**
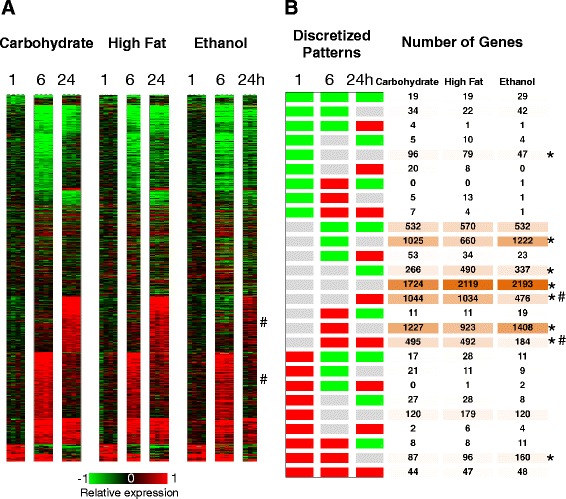
Differential gene expression patterns in response to PHx in the three dietary groups. **a** Average differential expression was computed at each time point post PHx, based on comparing PHx samples and the corresponding excised LLM tissue at *t* = 0 from the same animals. The genes are ordered based on the expression patterns in the Carbohydrate group. Coarse-grain visualization of the differential expression at this scale suggests a broadly similar dynamic patterns of response in all the three dietary groups. The specific differences are explored systematically using a COMPACT matrix (Fig. 6). Of note are the significantly lower upregulation at 24 h in the Ethanol group (highlighted by the asterisks). **b** A total of 27 discretized expression patterns based on three possible levels of regulation (up, down and no change) at each of the three time points (1, 6 and 24 h post PHx). The pattern counts are based on a differential regulation threshold of 1.75 fold. There appears to be a broad similarity of dominant patterns across the three dietary groups. Of note are the significantly fewer genes in the Ethanol group for the dominant patterns corresponding to 24 h upregulation (highlighted by the # symbol). (two-tailed Z-test *p-value <0.0002)

One systems level question we addressed was whether adaptation to chronic ethanol intake alters the distribution of genes across the 27 expression patterns. We applied the dynamic response pattern analysis approach (Methods) to classify the differential gene expression in the three dietary groups along the 27 patterns (Fig. 5a). For a majority of the patterns, the number of genes was similar across the three dietary groups. Notable exceptions across the dietary groups were patterns with a 24 h differential response in which the Ethanol group had significantly fewer gene members (Fig. 5b). To assess the significance of differences in the gene counts between the ethanol and control groups, we compared the proportions of genes in the two diet groups using a two-tailed Z-test. A total of ten patterns were significantly different in proportion between the two groups (* *p*-value < 0.0002; Fig. 5b). The deficiency of differential regulation at this time point is likely to alter the proliferative response, as these sets were enriched for cell cycle and proliferation related processes in the control groups (Fig. 4c).

**Fig. 6:**
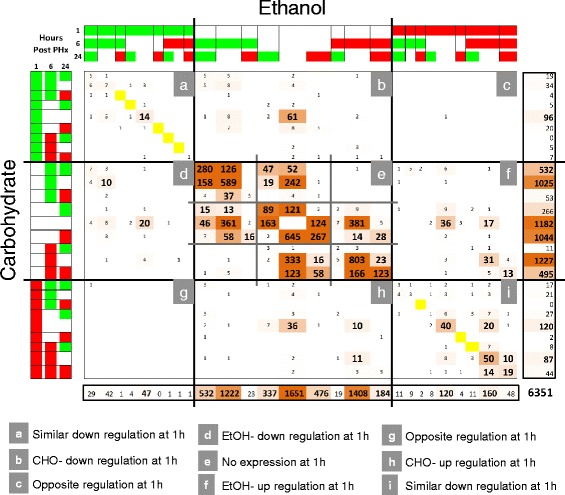
A 27 × 27 Comparative Pattern Count (COMPACT) matrix representing pair-wise counts of differential gene expression patterns comparing Ethanol and Carbohydrate groups. First, the differential gene expression at each time point was computed relative to appropriate, likely time point specific, control conditions. The time series data was averaged across replicates within each sample group, and discretized into up-(red), down-(green), and no-regulation (white) based on a threshold (1.5) of differential expression level. The sample groups were divided into two sets based on the comparative pair: Disease versus Normal. Within each set, the discretized time series expression data was collated for each gene into a pattern vector. Pairs of diet groups were compared to count the number of genes that follow each of the 27 * 27 (=729) possibilities to create a 27 × 27 matrix representing the comparative dynamic response pattern counts. The elements of the COMPACT matrix are based on pair-wise gene counts of the corresponding patterns, i.e., the element at the i^th^ row and j^th^ column of the matrix contains the number of genes that show an i^th^ expression pattern in Carbohydrate group and j^th^ expression pattern in Ethanol group. The diagonal of the matrix (yellow) represents those genes showing a common response and the off-diagonal elements of the 27 × 27 matrix represent the genes showing an altered temporal response between the two comparative conditions. The matrix was partitioned such that the pattern representing no differential regulation at 1 h broadly separates the up-and downregulation in the early response to PHx at 1 h. This yielded 9 sections corresponding to pair-wise combinations based on differential regulation at 1 h. The dominant patterns in section **e** reveal that the majority of the gene expression response occurred at 6 h and 24 h, but not at 1 h post PHx. The difference in gene counts in this section is highlighted using white (smallest) to dark brown (largest) set. Sections **c** and **g** corresponding to opposite regulation at 1 h between the dietary groups were nearly empty. Sections **b**, **d**, **f** and **h** correspond to genes that showed 1 h response only in one of the dietary groups. Sections **a** and **i** correspond to similar direction of regulation at 1 h, with the off-diagonal counts in these sections corresponding to genes with ethanol-altered patterns of regulation at 6 h and 24 h. Section **e** can also be subdivided based on differential regulation at 6 h, and showed similarities in overall structure, for example, with the largely empty anti-diagonal subsections (**c** and **g**) corresponding to opposite regulation at 6 h

We compared the Ethanol and Carbohydrate groups using our novel COMPACT matrix approach (Fig. 6). We analyzed the structure of the COMPACT matrix for various thresholds of discretization (Additional file 2: Figure S1, Additional file 3: Figure S2, Additional file 4: Figure S3, Additional file 5: Figure S4, Additional file 6: Figure S5, Additional file 7: Figure S6 and Additional file 8: Figure S7). At a very high threshold (fold change > 4), only the largest differential gene expression changes remained and populated a few elements of the COMPACT matrix (Additional file 2: Figure S1). As one continued to reduce the stringency, more elements of COMPACT were populated (compare Additional file 2: Figure S1, Additional file 3: Figure S2, Additional file 4: Figure S3, Additional file 5: Figure S4, Additional file 6: Figure S5, Additional file 7: Figure S6 through Additional file 8: Figure S7, in that order). However, beyond a threshold, the sparse structure remained largely the same, without significantly adding to the insights that can be gained from COMPACT analysis. In the subsequent analysis, we chose a threshold of 1.5 that allowed us to gain sensitivity in detecting the ethanol altered patterns at the 1 h time point. Analysis of the COMPACT matrix revealed a significant number of off-diagonal elements indicating many gene expression alterations in the Ethanol group (4050 genes out of 6351 total). The non-zero elements of the COMPACT matrix were sparsely distributed with only 35 comparative patterns with 20 or more genes (201 non-zero comparative patterns, out of 729 total). A total of 34 out of 35 largest comparative patterns were present in the sections along the principal diagonal (sections a, e, and i of the COMPACT matrix, Fig. 6), i.e., there were relatively few genes whose response was altered from up- to downregulation and vice versa due to ethanol treatment compared to the Carbohydrate control (187 genes sparsely distributed in the sections along the anti-diagonal) (sections c and g of Fig. 6). The dominant patterns in section (e) reveal that the majority of the gene expression response occurred at 6 h and 24 h, but not at 1 h post PHx. This section was almost entirely structured along the diagonal indicating that very few genes show opposing responses between Ethanol and Carbohydrate groups, similar to the overall structure of expression patterns found in the immediate early response. This indicates that switching between upregulation and downregulation is not a major component of the overall effect of ethanol intake on response to PHx. The structure of the COMPACT matrix focused on comparing the Ethanol and High Fat groups is included in the Additional file 9: Figure S8.

As a comparison to an unsupervised clustering approach, we considered WGCNA for identifying gene co-expression clusters [5]. WGCNA computes gene expression correlation and uses a threshold to detect modules that are subsequently merged based on an eigen-gene analysis that identifies representative gene expression patterns based on PCA. WGCNA identified 16 gene co-expression modules that were subsequently merged into 5 modules at a co-expression threshold of 6 and similarity threshold of 0.25 for module merging (Additional file 10: Figure S9). We exhaustively enumerated the intersections between the WGCNA module membership and COMPACT groups and represented the results using a COMPACT matrix-like structure (Additional files 11: Table S2 and Additional files 12: Table S3). Our analysis indicated that there was not a direct one-to-one correspondence between the WGCNA and COMPACT groups. However, the dominant patterns identified by COMPACT were largely within individual WGCNA modules. The most notable difference was that several COMPACT groups with relative low counts were contained within the larger WGCNA modules, and were not as readily distinguishable.

A total of 26 out of the 35 largest comparative patterns in Fig. 6 were based on a lack of immediate early response at 1 h, as highlighted in the central square of the COMPACT matrix in Fig. 6. (19 of the 20 largest comparative patterns, and 5611 of 6351 genes were in this square). These results reveal that the dominant aspects of ethanol-dependent gene expression alterations occur during the transitional and replicative phases of liver regeneration between 6 to 24 h post PHx. We explored these dominant patterns as well as relatively subtle immediate early patterns as detailed below. In the following analysis, the term “novel” corresponds to a set of genes showing differential expression only in the Ethanol group but not in the Carbohydrate control group. Similarly, the term “missing” corresponds to genes that do not show differential expression in Ethanol group, but are differentially regulated only in the Carbohydrate control. The term “common” is used to denote the genes with similar patterns of differential expression between the two dietary groups.

### Ethanol adaptation induces aberrant immediate early response to partial hepatectomy

**Fig. 7:**
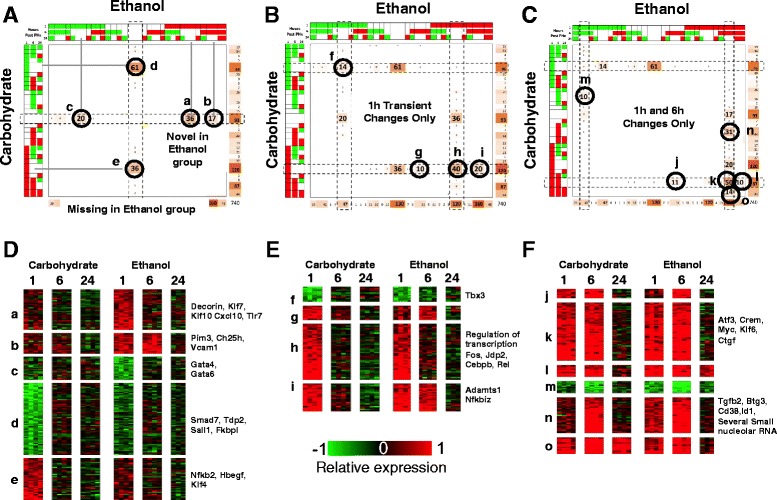
Analysis of a subset of the overall COMPACT matrix comprised of comparative patterns corresponding to differential regulation at 1 h in either dietary group. A set of 740 genes was distributed across the selected subset of patterns. **a** Differentially regulated genes that are novel or missing in the ethanol group are highlighted respectively in the middle column and row of the COMPACT matrix. Of the 93 genes with ethanol-specific response patterns, 36 showed transient upregulation, 20 showed transient down regulation, and 17 showed persistent up regulation until 6 h. Select key genes in these sets are indicated in D. Of the 130 genes with differential response that are missing in the ethanol group, 61 genes showed transient down regulation and 36 genes showed a transient up regulation at 1 h in the carbohydrate control group. **b** Comparative patterns corresponding to 1 h transient differential regulation in either dietary group are highlighted. The dominant patterns corresponded to gene sets with similar transient up or down regulation (sets f and h, respectively), as well as gene sets with ethanol-altered pattern of expression with shift towards differential regulation at 6 h (sets g and i). **c** Comparative patterns corresponding to 1 h and 6 h changes only (i.e., no differential regulation at 24 h) in at least one dietary group are highlighted. The dominant comparative pattern with 50 genes corresponded to similar direction of up regulation between the dietary groups at 1 h and 6 h. Other comparative patterns with more than 10 genes are also highlighted, corresponding to ethanol-altered differential regulation. **d**, **e**, **f** Heat map representation of gene expression patterns corresponding to the highlighted gene sets from panels **a**, **b**, and **c**, with select key genes indicated in each set

We initially focused on the immediate early response at 1 h when the gene expression changes were relatively less extensive. We masked the central section of the COMPACT matrix and highlighted the dominant patterns with the immediate early response (Fig. 7). A total of 17 out of 648 comparative patterns (=729 total – 81 in central section) contained 10 or more genes. The majority of the responses in the Carbohydrate and Ethanol groups were distributed along four patterns (transient up-or downregulation at 1 h, upregulation at 1 h that is persistent at 6 h only, or at both 6 h and 24 h). The novel and missing response patterns in the Ethanol group were largely distributed along the 1 h transient with a notable exception of 17 genes that showed a novel persistent response that was lacking in the controls (Fig. 7a).

### Novel/missing in ethanol diet

A large fraction of the 1 h transient differential regulation response was lacking in the Ethanol group (Fig. 7a; 61 downregulated genes, 36 upregulated genes). There were several novel responses in the Ethanol group (36 upregulated, 20 downregulated) with notable genes such as Decorin, *Klf7*, *Klf10*, *Cxcl10*, *Tlr7*, *Cd86*, Thrombin receptor, platelet factor 4, in the upregulated set (Fig. 7d). These sets included key genes such as *Hbegf*, *Klf4* and *Nfkb2* in the lack of upregulation and *Smad7*, *Ttrap*, *Sall1*, *Fkbpl*, in the lack of downregulation.

### Transient at 1 h

A total of 40 immediate early genes showed a common response between the Ethanol and Carbohydrate groups (Fig. 7b), with similar magnitude of differential regulation between the groups. This set included key regulators such as *Fos*, *Jdp2*, *Cebpb*, and *Rel* (Fig. 7e). Fos is a component of the AP-1 family of regulators and is a widely observed characteristic signature of immediate early response [53, 54]. JDP2 functions as a dominant negative factor by binding to c-JUN (another member of AP-1 family) and inhibiting the transcriptional regulatory activity [55]. The observed upregulation of *Jdp2* is likely relevant as controlling the duration of AP-1 activity in the early phase of the regenerative response. NF-kB activation in the Kupffer cells, followed by hepatocytes, is a well-described immediate early response to partial hepatectomy, and is critical to initiate transitional processes during the early phase of liver regeneration [24]. Our results implicate concomitant gene expression changes in this regulator. C/EBP-beta is critical to driving the hepatocyte response to liver regeneration [21, 56]. *Cebpb* knockout mice show a significantly blunted regenerative response correlating with altered expression of cell cycle genes [21].

A set of 20 genes was altered in the Ethanol group to remain persistently upregulated at 1 h and 6 h (Fig. 7b). This set included genes such as *Adamts1* and *Nfkbiz* (Fig. 7e). HB-EGF has been shown to be critically required in transitioning to late G1 phase [57]. SMAD7 is an inhibitor of SMAD2/3 activation by TGF-beta. Lack of downregulation of *Smad7* in the Ethanol group might lead to inhibited or reduced activation of SMAD2/3 during liver regeneration. While a role for SMAD3 in the early and transitional phases of liver regeneration remains to be characterized, it has been speculated that SMAD3 activity is required to suppress inhibitors of differentiation genes, *Id1*, *Id2*, and *Id3* [13]. In our results, *Id1* showed an early and persistent upregulation in the Ethanol group, but only a transient increase at 6 h in the Carbohydrate group (Fig. 7f). *Id2* and *Id3* were upregulated at 6 h by 2-fold in Carbohydrate group alone, and *Id2* was downregulated at 24 h by >2-fold in both groups.

NF-kB2 p100 plays a key role in down regulating the NF-kB transcriptional regulatory activity by inhibiting NF-kB p65 via dimerization [58]. TDP2 (also referred to as TTRAP, Traf and Tnf receptor associated protein) has been shown to associate with TNF receptor to inhibit activation of NF-kB [59]. Lack of downregulation of *Tdp2*, lack of upregulation of *Nfkb2*, and persistent upregulation of *Nfkbiz* in the Ethanol group indicates an aberrant NF-kB activity in the early and transitional phases of regeneration. Understanding the integrated impact on NF-kB activity requires further characterization of relative contributions of the inhibitory factors and potential cell-type specific differences between parenchymal and non-parenchymal cells.

### Persistent at 1 h and 6 h

A total of 50 genes showed a common persistent upregulation in the Carbohydrate and Ethanol groups (Fig. 7c). Of these genes, a set of 15 genes showed higher magnitude of upregulation at 6 h in the Ethanol group compared to the Carbohydrate group. The set of 50 genes included regulators such as *Atf3*, *Crem*, *Myc* and *Klf6*, and *Ctgf*, *Gadd45a*, and *Got1* (Fig. 7f). CTGF has been shown to decrease the availability of SMAD7 and increase SMAD2 activity in kidney tubule cells [60] and hence promotes TGF-beta signaling. However, *Smad7* was downregulated in Carbohydrate but not in Ethanol group (Fig. 7d, detailed above). In contrast, *Gdf15* was similarly upregualted transiently at 1 h in Ethanol and Carbohydrate groups. GDF15, also known as MIC-1 is a member of TGF-beta superfamily known to counteract CTGF by direct binding [61] and to inhibit hypertrophy via activation of SMAD2/3 [62]. *Gdf15* is expressed only by hepatocytes within 30 mins after PHx and normalized to basal levels by 2 h, with potential redundant functions during liver regeneration [63].

A group of 31 genes showed an altered response that was immediate early and persistent in the Ethanol group, but showed a transient response only at 6 h in the Carbohydrate group (Fig. 7c). This set included genes such as *Tgfb2*, *Btg3*, *Cd38*, *Id1* (Fig. 7f), and several Small nucleolar RNA. *Cd38* is constitutively expressed in the hepatic stellate cells and is upregulated upon activation, inducing *Il6*, and adhesion molecules [64]. Consistent with these results, *Vcam1* showed a persistent upregulation at 1 and 6 h in the Ethanol group, but did not change in the Carbohydrate group.

### Ethanol effects on gene expression changes during the transitional and replicative phases reveal deficiencies in system-wide transcriptional programs

We extended our findings from the immediate early response to later time points during the transitional (6 h) and replicative (24 h) phases. We employ the term “transitional” to refer to the 6 h time point, when cells are transitioning into the early G1 phase, as an intermediate phase separating the immediate early 1 h time point and the 24 h time point with the ongoing cell cycle [24]. In rats, the hepatocytes are in the peak DNA synthesis phase at 24 h following PHx, and hence we refer to the 24 h time point as “replicative” phase in the following analysis. In order to explore the systems-level gene expression impacted by chronic ethanol intake in the transition and replicative phases, we focused on the subset of overall COMPACT matrix corresponding to gene expression response only at later time points, 6 h and 24 h (section e of Fig. 6). This matrix was less sparse than that of the immediate early response at 1 h. However, the lack of population along the anti-diagonal indicates that even in the transitional and replicative phases, the systems level deficiencies in gene expression changes in the Ethanol group largely are not characterized by switching between activation and inhibition compared to Carbohydrate controls.

**Fig. 8:**
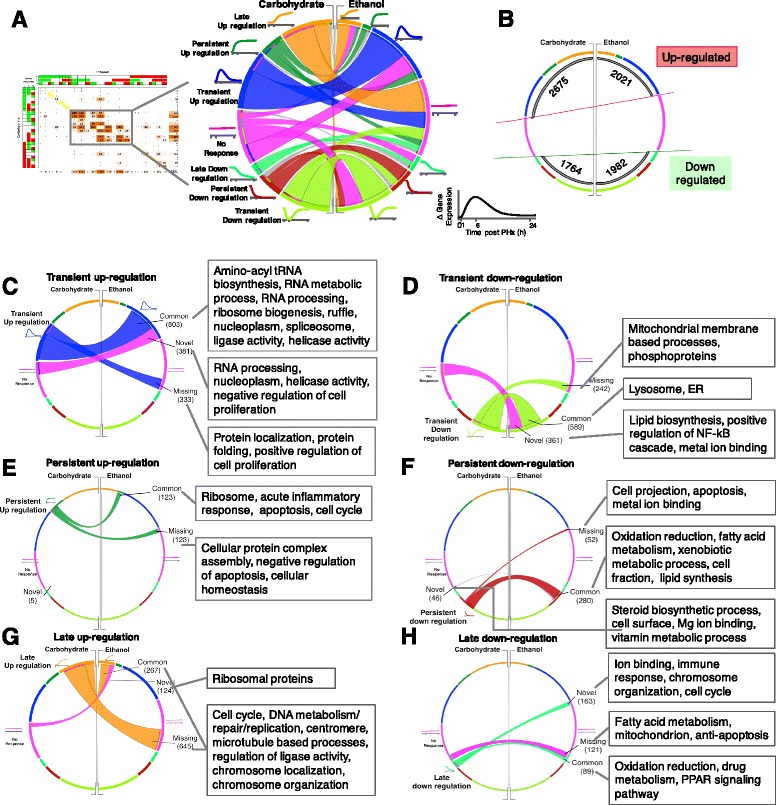
Analysis of comparative patterns corresponding to differential regulation at 6 h and 24 h, but not earlier at 1 h. A set of 5611 genes was distributed across the selected subset of patterns from section e of the overall COMPACT matrix shown in Fig. 7. **a** Circular representation of the comparative patterns using the CIRCOS tool. The length of the arcs is proportional to the number of genes showing the corresponding pattern. The width of the ribbons connecting the arcs corresponding to the comparative pattern count, i.e., the number of genes showing the corresponding differential regulatory patterns in the two dietary groups. The arcs are arranged such that the no-differential-regulation pattern separates the upregulated patterns (upper segment) and the down regulatory patterns (lower segment). Absence of dominant ribbons crossing the two segments demonstrates that relatively few genes switch the direction of PHx-induced differential regulation between the dietary groups. **b** Ethanol group shows a lower number of up regulated genes (2021) than the Control group (2675), as indicated by the net sum of corresponding arc lengths. In comparison, the difference was lower in the total down regulation between the Ethanol (1982) and Carbohydrate (1764) groups. **c**-**h** The dominant gene sets within the six differential expression patterns are highlighted: **c** transient upregulation, **d** transient downregulation, **e** persistent upregulation, **f** persistent downregulation, **g** late upregulation, and **h** late downregulation. In each set, a select list of statistically over-represented functional annotation information is indicated

We utilized a chord diagram representation of the transition and replicative phase COMPACT matrix to guide our subsequent exploration of systems-level gene expression changes (Fig. 8a). We utilized the Circos software for this purpose [65]. While Circos has been largely used to visualize genomic and other sequence datasets, e.g., genomic synteny, structural variation, next-gen read sequence mapping, etc., it has been recently used for visualization of generic tables in a few instances [66]. We adapted this functionality to develop a structured statistical graphic to highlight the underlying systems-level organization of the gene expression results (e.g., as illustrated in Fig. 8). In this visual, the segmented arcs of a circle correspond to the individual expression patterns (i.e., rows and columns of COMPACT matrix), with the length of an arc segment proportional to the number of genes exhibiting the corresponding expression pattern. We organized the Comparative Pair across the ‘meridian’ to readily enable parallel comparison of individual as well as cumulative pattern sizes (based on arc lengths). Within each dietary group, the arcs for up- and downregulatory expression patterns were separated by a null pattern representing ‘No differential expression’. This enabled easy identification of any gene sets that switched from up-to downregulation between the groups in the Comparative Pair, as these would appear as ‘ribbons’ crossing the ‘equatorial’ midline area. This representation readily highlights the overall deficiency of upregulation in the Ethanol group (compare the cumulative length of left vs. right arcs in top and bottom halves of Fig. 8b). Within the upregulation patterns, the Ethanol group had significantly fewer genes showing late or persistent upregulation (compare left vs. right arc lengths at the same level in Fig. 8a). The overall downregulation is subtly higher in the Ethanol group without major differences in the individual patterns.

Within each dynamic pattern, the majority of genes fell into one of four pairwise comparative patterns: unique to one or the other dietary group, or common to both groups, or persistent regulation in one group turning to a transient one in the other group. The last feature or aberrant temporal activation/inhibition was more prevalent in the downregulation patterns than in the upregulation patterns. Interestingly, there were very minor fractions of genes that showed a temporal shift in response from 6 h transient to 24 h late differential regulation between Ethanol and Carbohydrate groups (note the relatively little connectivity between corresponding patterns in Fig. 8a).

These results based on overall pattern counts indicate that chronic ethanol effects are mediated by a deficiency in overall activation signals in the regulatory networks driving liver regeneration. However, as we examined the representative processes in these groups we found that the Ethanol group shows deficiencies as well as novel regulation in contrasting aspects of key pathways, as detailed below.

### Transition phase (6 h) expression patterns reveal deficiencies and alterations in multiple pathways due to ethanol treatment

A majority of genes with a transient expression at 6 h were common between the Ethanol and Control groups (803 genes). Of these, a set of 96 genes showed larger magnitude of upregulation in the Ethanol group than in the Control group. However, a substantial number of genes were missing (333 genes) or showed novel upregulation (381 genes) in the Ethanol group (Fig. 8c). The common 803-gene set was enriched for processes including cell cycle (particularly mitotic phase); chromosome organization; biosynthesis of tRNA, protein and ribonucleoprotein complex; helicase activity; cell projection; MAPKKK cascade; and, leukocyte migration. The missing response included processes such as positive regulation of cell proliferation; chaperone mediated protein folding; immune cell effector processes including leukocyte activation and proliferation; positive regulation of cytokine (including interferon gamma) production; lipid biosynthetic process; positive regulation of cell differentiation; and, regulation of apoptosis. In contrast, the Ethanol group showed novel upregulation of 381 genes involved in processes such as negative regulation of cell cycle, helicase activity, RNA processing (particularly via spliceosome), and tRNA metabolic and acetylation process. We found an interesting contrast in which upregulation of genes involved in “positive regulation of cell proliferation” was missing in the Ethanol group, whereas genes involved in “negative regulation of cell proliferation” were uniquely upregulated in the Ethanol group (Fig. 8c). These results demonstrate a coordinated upregulation of inhibitors coupled with a lack of upregulation of activators as underlying the inhibition of proliferation in the Ethanol group as early as 6 h during the transitional stage of liver regeneration.

As similar processes were representative of the gene groups unique to one of or common to both Ethanol and Carbohydrate groups, we further investigated the specific genes in the deficient or aberrant upregulation set in the Ethanol group (Fig. 8d), While genes involved in immune cell migration showed an upregulatory response common to Ethanol and Carbohydrate groups, the genes driving immune cell proliferation (interleukins *Il7* and *Il13*, *Cxcr2*, *Csf1*, *Btc* betacellulin, *Nbn*, *Jnk2*, etc.), as well as in positive regulation of cytokine production (*Jnk2*, *Gata3*, *Bcl3*, similar to *Hsp60*) were uniquely upregulated only in the Carbohydrate controls. Six genes involved in lipid biosynthetic process were upregulated only in the Carbohydrate controls. Several genes corresponding to the protein folding function were upregulated in the Carbohydrate group but not in the Ethanol group. In agreement with the earlier studies of ethanol induced ER stress [67, 68] these results indicate that the Ethanol group may not have sufficient capacity to appropriately process the protein products downstream of translation, which could further exacerbate the deficiency in regenerative response.

We further visualized the novel, common and missing regulation of the set of genes that showed persistent regulation. Both persistent upregulation (Fig. 8e) and downregulation (Fig. 8f) showed a relatively low novel response. Notable is the presence of a set of anti-apoptosis/apoptosis related genes that is missing in the both upregulated/downregulated set of genes in the Ethanol groups. Genes involved in homeostasis showed missing responses in persistent upregulation and we found a set of cell cycle genes (*Gadd45a*, *Myc*, *Tgfb3*) showing a common response. While the common set in persistently downregulated genes showed association with important functions like oxidation-reduction, drug metabolism and lipid synthesis, late (24 h) up regulation showed a substantial number of missing genes (645) participating in crucial functions like cell cycle and DNA metabolism (Fig. 8g) Late down regulation showed relatively more novel (163) genes compared to missing (121) with 89 genes common between the diet groups (Fig. 8h). We analyzed these gene sets further for functional pathways and putative cell type specific contributions, as discussed below.

### Replicative phase (24 h) expression patterns reveal deficiencies and alterations in the cell proliferation/cycle pathways due to ethanol action

**Fig. 9:**
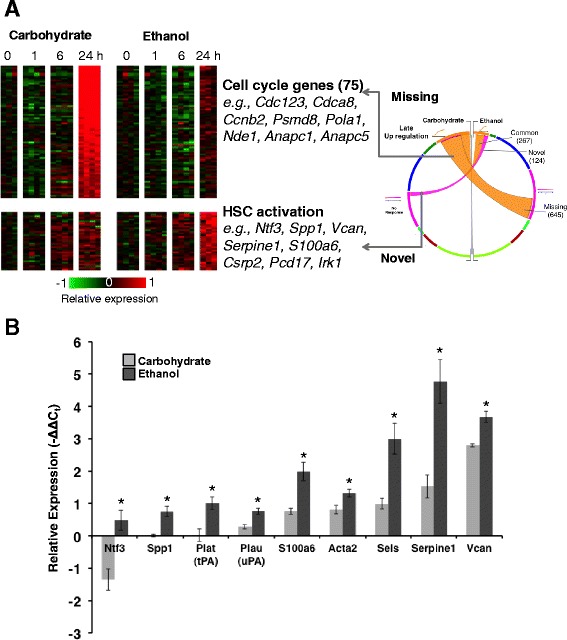
Analysis of comparative patterns corresponding to up regulation at 24 h, but not earlier at 1 h and 6 h. **a** The expression patterns of gene sets with missing and novel differential up regulation are shown in the heat map. Select key genes in each set are indicated. **b** Real-time PCR based evaluation of differential expression of genes corresponding to HSC activation state at 24 h post PHx.–∆∆Ct values are shown as compared to *Gapdh* expression within each sample, and compared to the corresponding LLM paired-samples within each dietary group. *N* = 3 biological replicates. Error bars: +/− standard error of the mean. * *p* < = 0.05)

Ethanol effects on gene expression are most evident in the response at 24 h with 645 genes (largest element of the COMPACT matrix) showing no differential expression in the Ethanol group (Fig. 9a). This set contained 75 genes involved in cell cycle (particularly the mitotic phase). For example, some of the mitotic cell cycle related genes included *Cdc123*, *Cdca8*, *Ccnb2*, *Psmd8*, *Pola1*, *Nde1*, *Anapc1* and *Anapc5*. Other functions included those driving chromosome organization (kinetochores) (*Dsn1*, *H2afz*, *Cenp1*, *Cenpa*, *Cbx5*), microtubule-based processes such as kinesins (*Kif11*, *Kif15*, *Kifc1*, *Kif2c*, *Prkcz*), DNA metabolic process and DNA repair (*Mgmt*, *Psip1*, *Cdc7*, *Mdc1*, *Tdp1*, *Rpa3*, *Rfc4*), replication fork members (*Dnmt1*, *Pola1*, *Pola2*) etc. A set of 267 genes showed upregulation in Ethanol and Carbohydrate groups, however the magnitude of the response is significantly lower in the Ethanol group. This set contained genes involved in functions such as ribosome (*Rps19*, *Rps16*, *Rps15*, *Rps12*), chromosome organization (*Prim1*, *Hist1h2bm*, *Mcm2*, *Rfc3*), cell division (*Cdc2*, *Nuf2*, *Anln*, *Pttg1*, *Ccnb1*), cell cycle related (*Smc2*, *Mad2l1*, *Plk1*), DNA replication (*Roa2*, *Ccne1*, *Rfc3*, *Mcm7*, *Rrm1*, *Pold2*) and DNA metabolic processes. Differential expression of the set of genes at 24 h post PHx in our data is also largely reflective of the replicative stage of the cell cycle. This is consistent with earlier expression studies on liver regeneration showing that genes induced at 24 h are cell cycle genes and are involved in DNA replication and chromosomal organization [69]. Our data is also in agreement with the previous work showing that chronic ethanol treatment inhibits cell proliferation in the mouse as it does in the rat [36], specifically cell cycle progression through S phase (which peaks at 22–24 h. in the rat). Our gene expression signature at 24 h post PHx demonstrated robust cell cycle induction in the control diet compared to the Ethanol group. We speculate that the negative regulation of these replicative genes could contribute to inhibition of regeneration. Recent studies emphasized the importance of the contribution of multiple cell types and microRNAs to the damage repair mechanisms of the liver after chronic ethanol intake [70]. We investigated the cell type specific response by exploring differentially expressed genes for cell type specific markers.

### Hepatic stellate cell response mediated by ethanol intake at 24 h post PHx

A set of 124 genes showed a novel upregulation in the Ethanol group at 24 h post PHx. Our initial pathway analysis showed enrichment in broader functional categories such as structural constituent of ribosome (*Rps26*, *Rps28*, *Rpsa*), intracellular protein transport (*Tmed2*, *Fxcl*, *Fxcl*-*ps1*, *Gosr2*, *Vcan*, *Sels*) and protein biosynthesis (*Eef1g*, *Ehd2*, *Eef1b2*). The initial round of hepatocyte replication during liver regeneration is followed by proliferation of the non-parenchymal cells by approximately 48 h [23, 71]. However, the effect of ethanol intake on such selective activation is still a topic of research. It is known that chronic ethanol intake can lead to characteristic differences in cell type-specific responses [72, 73]. Closer inspection of the gene set revealed several hepatic stellate cell (HSC) specific transcripts showing aberrant up regulation, albeit with relatively weaker signals, in the Ethanol group (Fig. 9a). These genes include *Vcan* (versican), *Ntf3* (neurotrophin-3), *S100a6* (S100 calcium binding protein A6), *Spp1* (osteopontin), *Pcdh17* (protocadherin-17), *Csrp2* (cysteine and glycine-rich protein 2), *Cygb* (Cytoglobin), *Cbr1* (cannabinoid receptors type 1) and *Fkbp7* (FK506 Binding Protein 7). Recent studies have shown the presence and activation of these factors in HSCs. A few of these factors are established markers of HSCs, while others have been predicted using high throughput genomic and proteomic analysis. For example, *Cygb* is a known to be a marker for activated stellate cells in human HSCs [74]. Osteopontin, an inflammation marker, is one of the genes upregulated in cultured activated rat HSCs [75]. Analysis of human hepatic mRNAs from patients with progressive stages of ALD showed that inhibition of osteopontin-receptor mediated signaling partially inhibited ethanol induced HSC activation [76]. Other factors that are upregulated in cultured activated rat HSCs include Protein S100-A6 [77], Versican [78], Protein Fkbp7 [79] and neurotrophin [80]. In order to validate the microarray findings, we performed high throughout qPCR for several HSC functional state marker genes including *Acta2* (α-SMA), *Serpine1* (PAI-1) and *S100a6*. We found the results to be consistent with microarray data (Fig. 9b). One exception was *Acta2* (α-SMA) that showed upregulation in the qPCR analysis, which was not predicted as differentially regulated by the microarray data analysis. Taken together, our results uncovered a new finding on a putative role for HSC stimulation in ethanol-mediated suppression of the regenerative response following PHx.

## Discussion

We developed a novel comparative pattern analysis approach (COMPACT) to systematically and exhaustively evaluate the gene expression results to identify key gene groups that are altered by external perturbations. In the present study, we employed a ternary discretization of average gene expression (+1, 0, −1) for each diet group as an input to COMPACT analysis. However, the COMPACT approach is not limited to a ternary approximation (up, down, no change). For example, one can consider two levels of up or down (e.g., at two different thresholds), or unequal levels between up versus down, etc., to form the disecretized patterns. The “information loss” that may occur from discretization is balanced by the “information gain” in the exhaustive comparative grouping of the patterns. The core idea is to develop the COMPACT two-way histogram that can then be mined for key gene groups. The underlying and expected biological correlations in the data will be reflected in the sparse structure of the COMPACT matrix, guiding subsequent analysis. We utilized this novel approach to investigate the temporal gene expression changes in the rat liver due to chronic ethanol intake. We visualized and quantified systems level differences to elucidate changes in diet-specific gene expression during the time course of liver regeneration. Systematic parsing using this approach helped to identify the dominant alterations as responses to the ethanol diet, and by masking these prominent responses we were able to uncover the subtler gene expression patterns that were informative, for instance, on the non-parenchymal cellular responses.

The ethanol-adapted liver showed modest expression changes for a number of genes compared to control livers. The additional perturbation induced by PHx caused significant changes in the liver transcriptome. Ethanol adaptation induced an aberrant immediate early response to partial hepatectomy. However, we found that ethanol intake affects the complex process of liver regeneration, largely during the transitional and replicative phases, between 6 to 24 h post PHx. This was substantiated by the significant participation of replicative genes at 24 h post PHx. We also discovered differential expression of HSC specific genes in the Ethanol group samples at 24 h post PHx. We speculate that chronic alcohol consumption dynamically shifts HSCs into a distinguishable anti-regenerative activation phenotype post-partial hepatectomy, thereby altering the balance between pro-regenerative and anti- regenerative hepatic stellate cells.

Overall, the liver transcriptome showed a strong adaptation to chronic ethanol intake. Distinct clustering of the Ethanol group compared to the Carbohydrate and High Fat diet groups ruled out the possibility that this may be a result of the caloric response (Fig. 3a). However, the Ethanol group does not show hepatocyte proliferation unlike the robust proliferation seen in controls. In the adapted state, we observed ethanol-induced alterations in metabolic processes such as lipid and sterol accumulation. Metabolic genes are known to be regulated on the circadian time scale [81]. The robust and stable changes in lipid homeostasis driven by differentially regulated circadian genes may be altering the regulatory network and suppressing normal regenerative response, by disrupting the molecular circadian clock in the liver. Our findings are in agreement with recent studies exploring ethanol’s ability to influence circadian rhythm [46, 82, 83].

The initial stages of liver regeneration are tightly regulated with G0-G1 transition phase followed by proliferative S phase. The secondary perturbation introduced by PHx created broader changes to the gene expression landscape, independent of the diet group as demonstrated by the transition to higher magnitude of gene expression post PHx (Fig. 3a). Such an altered sensitivity is a characteristic defense response of the organ to external damage and has been studied under various conditions in rats and other species [16, 25]. Our analysis revealed that ethanol adaptation induces aberrant immediate early responses to PHx. We found that most of the immediate early genes were activated in both Control and Ethanol groups, while some of the genes that are activated to promote regenerative response were Control-specific. We speculate this reflects the early signs of a delayed regenerative response in the chronic ethanol adapted liver. Our results also pointed to potential changes in non-parenchymal cells, particularly in the ethanol group.

Cell cycle is the dominant theme in the replicative phase, as demonstrated by the change in mRNA levels for a number of genes important for cell cycle process altered by ethanol intake. Ethanol-mediated inhibition of liver regeneration is cell cycle dependent, with hepatocytes being most responsive to the ethanol-induced damage during the early G0-G1 phase [32]. Hence, the aberrant mistimed early and persistent upregulation within 1–6 h in the alcohol group can not be seen as a compensatory response, but may be originated by the lack of capacity to mount appropriately timed response. Ethanol intake drives liver to have an insufficient preparation for hepatocytes to enter the cell cycle. This may very well be an early indicator of later activation of anti-proliferative processes, followed by the significant deficiency in upregulation of gene expression during the replicative phase. Such favoring of anti-proliferative responses induced by the combined stimuli of ethanol and PHx may lead to the disruption of the strict regulation of hepatocyte proliferation at the early stages of regeneration. This is in agreement with previous findings that ethanol intake causes a delay in regeneration potentially by inhibiting cell cycle entry [33, 34, 36].

Our analysis revealed the putative contribution of non-parenchymal cells to compensate for the inhibition of hepatocyte replication. We utilized whole tissue samples in our analysis, restricting the ability to detect and localize gene expression changes occurring in NPCs. However, we overcame some of these limitations by exhaustively enumerating even the subtle alterations using our COMPACT approach. This yielded multiple temporal patterns that could be attributed to an NPC response, in particular that of HSC activation. We found changes in the expression pattern of members of the KLF family of transcriptional regulators that are known to play a role in the stem cell or progenitor response during development and differentiation [84, 85]. One interpretation of aberrant expression of KLF family members in the Ethanol group is as an indicator of potential progenitor-like response during the initiation phase. There has been evolving literature on the contribution of progenitors to liver regeneration. Recent studies point to a lack of progenitor contribution to liver regeneration in multiple injury models [86, 87]. However, there is evidence for a progenitor contribution to long-term homeostatic tissue renewal in the case of hepatocyte senescence [88]. Other studies suggest the existence of hepatocytes with progenitor cell properties [89]. Interestingly, a recent study revealed the existence of hybrid periportal hepatocytes that proliferate and replenish liver mass following chronic hepatocyte-depleting injuries [90]. Our results are consistent with other studies on the modest contribution of progenitor cells in response to PHx under normal conditions.

Our results point to HSC gene expression changes as underlying the regeneration deficiency in the Ethanol group. Activated HSCs contribute to regeneration through production of angiogenic and pro-proliferative factors such as HGF to stimulate hepatocyte proliferation, as well as by ECM remodeling [91]. It is known that in the case of ethanol-induced liver injury, there are characteristic differences in cell type-specific responses, including HSCs and KCs indicating that ethanol treatment affects the interactions between hepatocytes and NPCs that are essential for a coordinated and integrated repair response [73]. The nature of those interactions and the factors that mediate them are only partially understood and little is known as to how ethanol intake affects those interactions. It was previously shown that treatment with acetaldehyde increased the production of Collagen I, leading to HSC proliferation [72]. Delineating the regulatory changes induced by chronic ethanol intake in multiple liver cell types at different stages in the regenerative process requires cell-type targeted experiments. However, using our novel COMPACT analysis, we could identify gene expression changes that could serve as NPC response signatures at various stages of regeneration. We found cell type specific participation in the immediate early (1 h post PHx) and transitional (6 h post PHx) phases of regeneration indicating a participation of non-parenchymal cells such as HSCs in the early phase of regeneration. Early time markers in the Ethanol groups were, decorin at 1 h, *Npy*, *Col4a1*, *Fn1*, *Serpine1* (PAI-1) at 6 h post PHx and persistent upregulation of *Vcam1*. This is in agreement with the finding that chronic ethanol intake induced an imbalance in the immune response, resulting in overproduction of TNF-α in Kupffer cells leading to changes in SREBP-1 and PAI-1 expression in HSCs [92]. Interestingly, we found a strong signature for aberrant HSC regulation at 24 h in the Ethanol group samples (although a modest upregulation of this cluster was also evident in the Carbohydrate controls). We further validated our results for the activation of some of the known HSC markers in the Ethanol group at 24 h post PHx (Fig. 9b).

The significant degree of HSC activation occurring after PHx in the Ethanol group indicates that ethanol treatment could be modifying the interactions between hepatocytes and HSCs that are essential for the coordinated and integrated tissue repair response and may therefore be disrupting the switch that maintains the HSCs de-activated. Such a combinatorial mechanism may lead to a feedback loop where there is limited cell cycle entry and increased HSC activation, which increases the ECM production that causes a further increase in growth factor sequestration, limiting the hepatocytes from entering cell cycle. This suggests that adaptation to ethanol intake is leading to a regulatory state of the tissue that is mounting an excessive/mis-timed HSC response, which underlies the deficient regenerative response observed. We hypothesize that these HSC-specific gene activations, by altering the push-pull balance between pro-regenerative phenotype and that of an anti-regenerative molecular state, helps to fine-tune the proliferation-inhibitory response associated with ethanol adaptation. Utilization of computational modeling [93–95] can further help characterize and understand how dynamic alterations in microenvironment and HSC activation within the liver contribute to deficient liver regeneration.

Our novel COMPACT approach can be applied more broadly to analyze biomedical and clinical Big Data. The tool organizes the data in unique ways to enable a broad systems-level analysis as well as pinpoint key aspects for deep mining. In an unsupervised analysis, disease or treatment relevant differences involving subtle changes in small groups of co-regulated genes are difficult to identify, if not entirely impossible to find. COMPACT overcomes the limitations of typical differential analysis and clustering methods that yield several long lists without a systematic way to sort out how these are interrelated and where to prioritize follow-up analysis. COMPACT exploits the statistical and visual paradigms that have been successful in other Big Data analysis contexts and adapts them for analyzing typical large-scale data on transcriptomics, proteomics, regulomics, metabolomics, etc. For example, the patterns could be formed from any sample annotation, including time series, dose response, demographic groups, etc. The comparative groups would be selected as appropriate to the particular study, for example, treatment versus control, male versus female, disease versus normal, drug A versus drug B, etc. Depending on the goals of the analysis, the sample annotation used for pattern formation and comparative groups can be switched. For instance, comparing patterns of expression across sample groups between two time points. Additional extensions include formation of patterns based on multi-modal data sets in which portions of the pattern vector are based on discretized quantitative data from multiple data types, while a subset of the pattern vector could be based on categorical information (‘responder’ versus ‘non-responder’, etc.). Thus, the two-way histogram approach of COMPACT is highly generalizable to a wide range of data analysis contexts.

## Conclusions

We developed a novel method, termed COMPACT, to analyze global gene expression time series data to identify key response patterns to perturbations. We applied our approach to assess the effect of chronic ethanol intake on the global gene expression response of liver to acute injury. We found that PHx induces broad changes to the liver transcriptome even during the deficient regeneration caused by chronic ethanol intake. Our results provide new insights into the mechanisms underlying ethanol-induced suppression of the regenerative response. Several transcriptional regulators and genes corresponding to metabolic processes, RNA processing, inflammatory response, ribosome, chromosome organization etc. showed similar differential regulation in Ethanol and Control groups. COMPACT analysis identified key gene expression patterns that were altered by ethanol treatment, corresponding to several important regulatory pathways including deficient cell cycle induction and non-parenchymal cell activation. Our results yielded a novel prediction on the potential role of HSC response and activation process in driving the ethanol-mediated defective regeneration phenotype.

## Methods

### Animals and tissues

Adult Sprague–Dawley rats were held in a climate controlled, 12-h day/night cycle in accordance with accepted animal handling practices. Animals were fed using the Lieber-DeCarli pair-feeding model [96] in which rats were fed a nutritionally adequate liquid diet containing 36 % of total calories derived from ethanol for 5 weeks (Ethanol group), with the pair-fed calorie-matched littermate controls receiving liquid diets in which ethanol calories were replaced by maltose dextran (Carbohydrate group). Rats (275–350 g) were anesthetized and subjected to 2/3rd PHx by surgical removal of left lateral and median lobes (LLM) as previously described [19, 70]. The remnant liver was allowed to regenerate and the liver samples were harvested at 1, 6 and 24 h post PHx and harvested (Additional file 1: Table S1). The excised liver samples at *t* = 0 served as within-animal controls. Collected liver samples were freeze-clamped in liquid nitrogen-cooled aluminium clamps for preparation of tissue lysates. Total RNA was isolated using TRIzol reagent (Invitrogen, Carlsbad, CA) according to the manufacturer’s instructions. All animal studies were approved by the Institutional Animal Care and Use Committee (IACUC) at Thomas Jefferson University.

### Microarray hybridization and data acquisition

We employed the Affymetrix Rat Gene 1.0 ST arrays with ~25000 probe sets for obtaining the transcriptomic profiles from each liver sample at the Genomics Core Facility, Thomas Jefferson University, yielding a total of 72 arrays (*N* = 4 biological replicates per group). The hybridizations were performed in three separate batches on separate days due to throughput limitations. Each batch of hybridizations included 4 replicates of t = 0 controls from each of the three diet groups permitting batch effect removal analysis to minimize the impact of variability potentially arising from multiple processing batches. MIAME compliant microarray gene expression data from 72 arrays were deposited in the Gene Expression Omnibus database. Accession # GSE33785 no. [70].

### Data normalization and outlier removal

The data normalization and statistical analysis to identify differentially expressed genes were performed using Partek Genomics Suite (Partek Inc., St. Louis, MO). The raw gene expression data was normalized using the standard Robust Multichip Average (RMA) approach [97]. Principal Component Analysis revealed outlier samples and these were excluded in further study. The RMA normalization was repeated for the remainder of the arrays after removal of the outlier samples.

### Differential gene expression

The normalized data was analyzed using a mixed effects ANOVA that considered the following two variables and their interactions as fixed effects: (1) Diet (Ethanol, High Fat and Carbohydrate), (2) Time post PHx (0, 1, 6 and 24 h). The microarray batch (three separate runs, as reflected in the array scan date) was considered as the random effect to account for run-to-run differences across arrays. Differentially expressed genes were identified based on statistically significant effects of Time post-PHx, Diet or an interaction between these two factors. The raw *p*-values from ANOVA were corrected for multiple testing using the standard *q*-value approach that estimates the proportion of non-differentially regulated genes and hence improves the sensitivity of the analysis [4].

### PHx-responsive genes

The list of differentially expressed genes from ANOVA was filtered based on a minimum fold change threshold of 1.5 up or down regulation in response to PHx, at any of the three time points, in Ethanol, High Fat or Carbohydrate dietary group animals. The fold-change filtered differentially expressed genes were considered further in the clustering, Principal Component Analysis and Pathway Analysis. In the heat map visualizations of these data sets, hierarchical clustering using complete linkage and Pearson correlation similarity metric was performed using the MeV software [98]. Clusters of genes were identified based on the expression pattern across all experimental groups including individual biological replicates.

### Visualization of sample groups

The differential gene expression data was used in an established Principal Component Analysis (PCA) approach using the princomp function implemented in the Bioconductor libraries [99] for the R Project for Statistical Computing. The samples were annotated based on a combination of Diet and Time post PHx, yielding nine distinct sample groups.

### Dynamic response pattern analysis and COMPACT matrix

Within each dietary group, for each time point post PHx; the gene expression data was normalized by subtracting the average of the corresponding LLM samples. The average differential gene expression data for each of the PHx groups was discretized to three levels (+1, 0,−1) based on a fold change threshold of 1.5 up or down regulation. Within each diet group, this discretization yielded a dynamic response pattern vector for each gene, encoded by one of 27 possible ordered sets of the three levels: +1, 0, and − 1. Pairs of diet groups were compared to count the number of genes that follow each of the 27 * 27 (=729) possibilities; to create a 27 × 27 matrix representing the comparative dynamic response pattern counts (COMPACT). For a given COMPACT matrix of comparative conditions C1 and C2, the element at the ith row and jth column of the matrix contains the number of features that show an ith pattern in C1 and jth pattern in C2. This pattern count matrix was visualized by adapting the Table Viewer functionality of the CIRCOS software [65].

### Pathway analysis

The PHx-responsive Genes were analyzed for over-represented biological pathways, networks and other functional annotation using the DAVID software [100]. The list of genes on the Affymetrix Rat Gene 1.0 array was used as the background reference in DAVID. The gene expression clusters were considered separately in the DAVID analysis.

### Quantitative validation using high throughput qPCR

Total RNA was isolated from frozen liver samples (~100 mg) using TRIzol (Invitrogen, Carlsbad, CA) according to the manufacturer’s directions. RNA concentration was measured by ND-1000 (NanoDrop, Wilmington, DE). High throughput qPCR was performed following standard BioMark (Fluidigm, South San Francisco, CA) qPCR protocol. Briefly, 1.2 μg of total RNA was reverse-transcribed using EasyScript Plus cDNA Synthesis Kit (Applied Biological Materials, Richmond, BC) and cDNA was stored at − 20 °C. 100 ng of synthesized cDNA was pre-amplified for 12 cycles using TaqMan PreAmp Master Mix (Applied Biosystems). Primers were designed using Universal Probe Library Assay Design Center (Additional file 13: Table S4). qPCR was performed using BioMark Dynamic Arrays (Fluidigm) with 40 cycles of amplification (15 s at 95 °C, 5 s at 70 °C, and 60s at 60 °C). C_t_ values were calculated by the Real-Time PCR Analysis Software (Fluidigm) and software-designated failed reactions were discarded from analysis. Relative gene expression was determined by the ΔΔC_t_ method. Mrpl16, Ubqln1 and Idh3B were used as housekeeping genes. Statistical significance was assessed using a two-tailed Student’s *T* test with unequal variances to compare the PHx-induced differential gene regulation between the Ethanol and Control groups at the 24 h time point.

### WGCNA analysis

We used the WGCNA method [5] to identify modules of highly correlated genes. We used a soft threshold value of 6 to identify the initial modules, and used a dissimilarity threshold of 0.25 for merging the initial modules into the final set of gene co-expression modules.

### Availability of supporting data/additional files

The data sets supporting the results of this article are available in the Gene Expression Omnibus (GEO) repository (Accession number: #GSE33785) and are publicly available at www.ncbi.nlm.nih.gov (http://www.ncbi.nlm.nih.gov/geo/query/acc.cgi?acc=GSE33785).

## Additional files

Additional file 1: Table S1. Normalized gene expression data for the statistically significant genes from the LLM samples, corresponding to the ethanol-adapted state. (XLSX 320 kb) Additional file 2: Figure S1. A 27 × 27 Comparative Pattern Count (COMPACT) matrix comparing the Ethanol and Carbohydrate groups for a fold change threshold of 4.0. (TIF 2033 kb) Additional file 3: Figure S2. A 27 × 27 Comparative Pattern Count (COMPACT) matrix comparing the Ethanol and Carbohydrate groups for a fold change threshold of 3.0. (TIF 2079 kb) Additional file 4: Figure S3. A 27 × 27 Comparative Pattern Count (COMPACT) matrix comparing the Ethanol and Carbohydrate groups for a fold change threshold of 2.0. (TIF 2443 kb) Additional file 5: Figure S4. A 27 × 27 Comparative Pattern Count (COMPACT) matrix comparing the Ethanol and Carbohydrate groups for a fold change threshold of 1.7. (TIF 2840 kb) Additional file 6: Figure S5. A 27 × 27 Comparative Pattern Count (COMPACT) matrix comparing the Ethanol and Carbohydrate groups for a fold change threshold of 1.5. (TIF 3263 kb) Additional file 7: Figure S6. A 27 × 27 Comparative Pattern Count (COMPACT) matrix comparing the Ethanol and Carbohydrate groups for a fold change threshold of 1.3. (TIF 4337 kb) Additional file 8: Figure S7. A 27 × 27 Comparative Pattern Count (COMPACT) matrix comparing the Ethanol and Carbohydrate groups for a fold change threshold of 1.1. (TIF 5210 kb) Additional file 9: Figure S8. A 27 × 27 Comparative Pattern Count (COMPACT) matrix comparing the Ethanol and High Fat groups for a fold change threshold of 1.5. (TIF 3185 kb) Additional file 10: Figure S9. Analysis of the gene expression data using WGCNA. Gene dendrogram obtained by average linkage hierarchical clustering. Module assignments using the Dynamic Tree Cut as well as the module merging process are shown underneath the dendrogram. (TIF 1841 kb) Additional file 11: Table S2. The distribution of genes between the COMPACT groups and the WGCNA dynamic modules prior to merging in WGCNA. The column labels correspond to the module colors assigned by WGCNA. The row labels indicate the COMPACT group size, carbohydrate pattern and ethanol pattern. For example, the row label 0589-c0-10-e0-10 corresponds to a COMPACT group that contains 589 genes showing a downregulation only at 6 h time point in both the dietary groups. (XLSX 49 kb) Additional file 12: Table S3. The distribution of genes between the COMPACT groups and the WGCNA merging modules. The column labels correspond to the module colors assigned by WGCNA. The row labels indicate the COMPACT group size, carbohydrate pattern and ethanol pattern. For example, the row label 0589-c0-10-e0-10 corresponds to a COMPACT group that contains 589 genes showing a downregulation only at 6 h time point in both the dietary groups. (XLSX 47 kb) Additional file 13: Table S4. List of real-time PCR primers. (XLSX 36 kb)

## Abbreviations

COMPACT: comparative pattern count
HSC: hepatic stellate cells
PHx: partial hepatectomy
TF: transcription factor
