# Supplementary figures and images for: A novel comparative pattern analysis approach identifies chronic alcohol mediated dysregulation of transcriptomic dynamics during liver regeneration
Source: BMC Genomics. 2016 Mar 25;17:260. doi: 10.1186/s12864-016-2492-x (PMC4807561; doi:10.1186/s12864-016-2492-x)

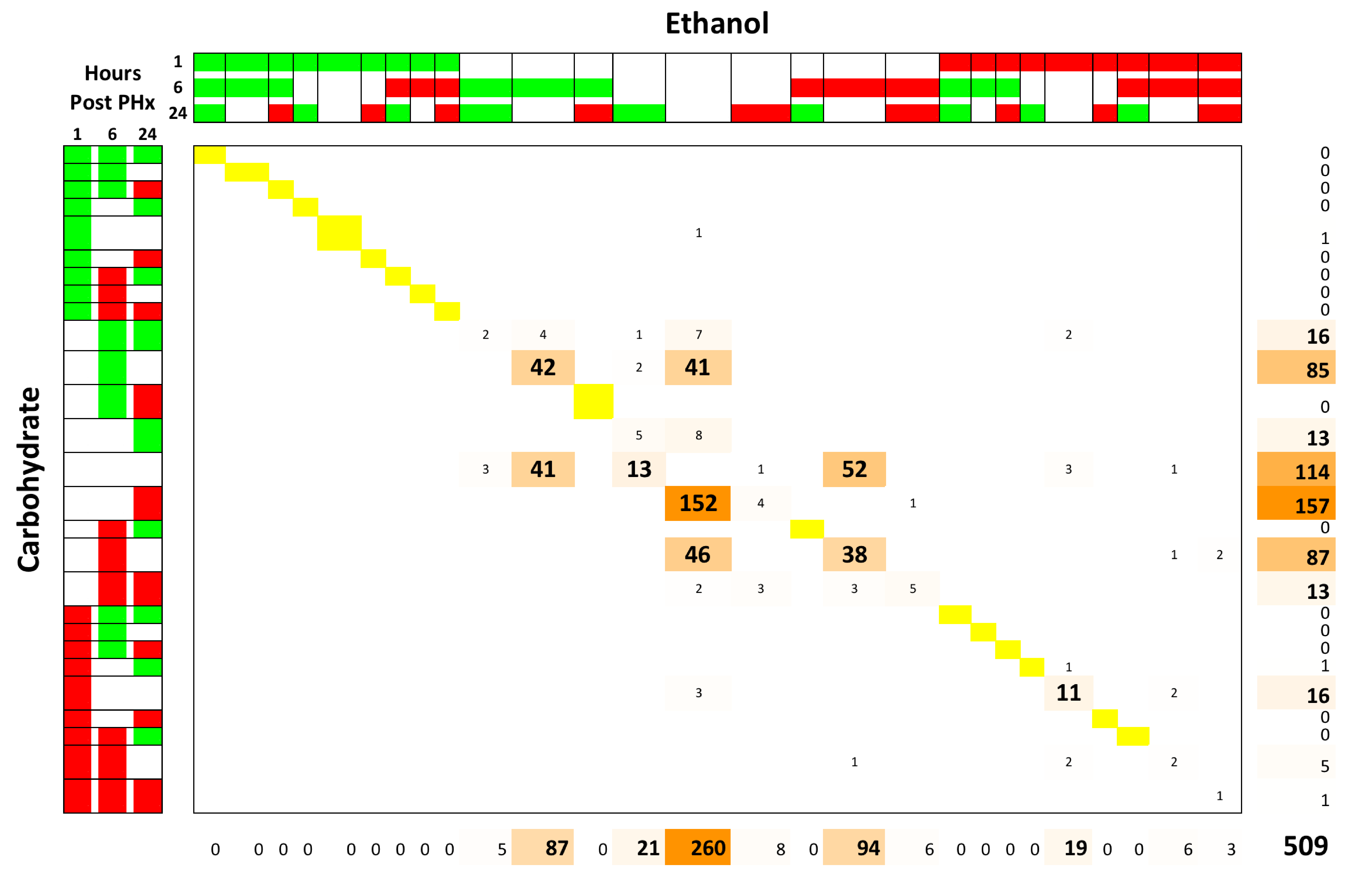

Supplement: Additional file 2: Figure S1. — A 27 × 27 Comparative Pattern Count (COMPACT) matrix comparing the Ethanol and Carbohydrate groups for a fold change threshold of 4.0. (TIF 2033 kb) [file 12864_2016_2492_MOESM2_ESM.tif]

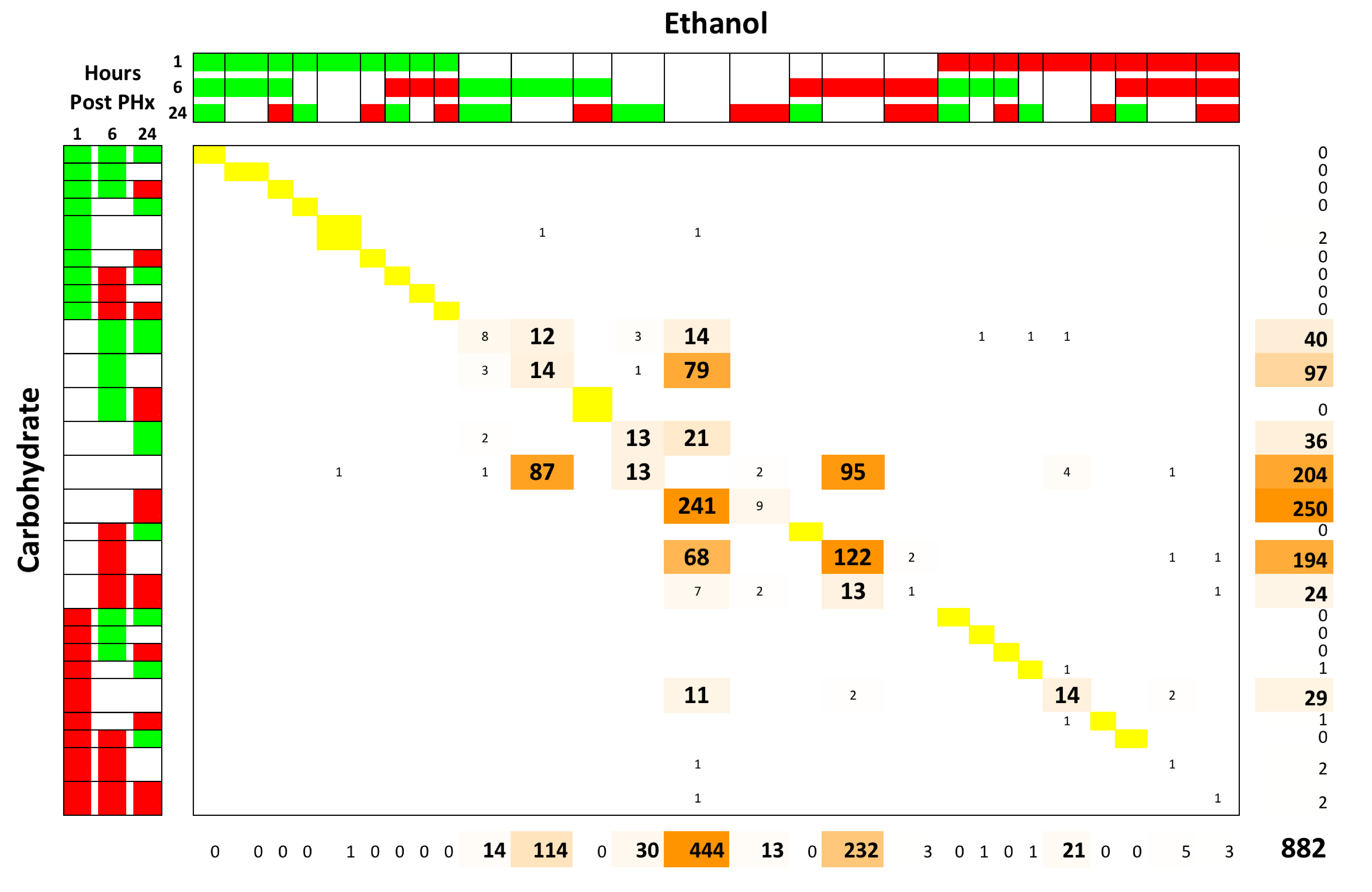

Supplement: Additional file 3: Figure S2. — A 27 × 27 Comparative Pattern Count (COMPACT) matrix comparing the Ethanol and Carbohydrate groups for a fold change threshold of 3.0. (TIF 2079 kb) [file 12864_2016_2492_MOESM3_ESM.tif]

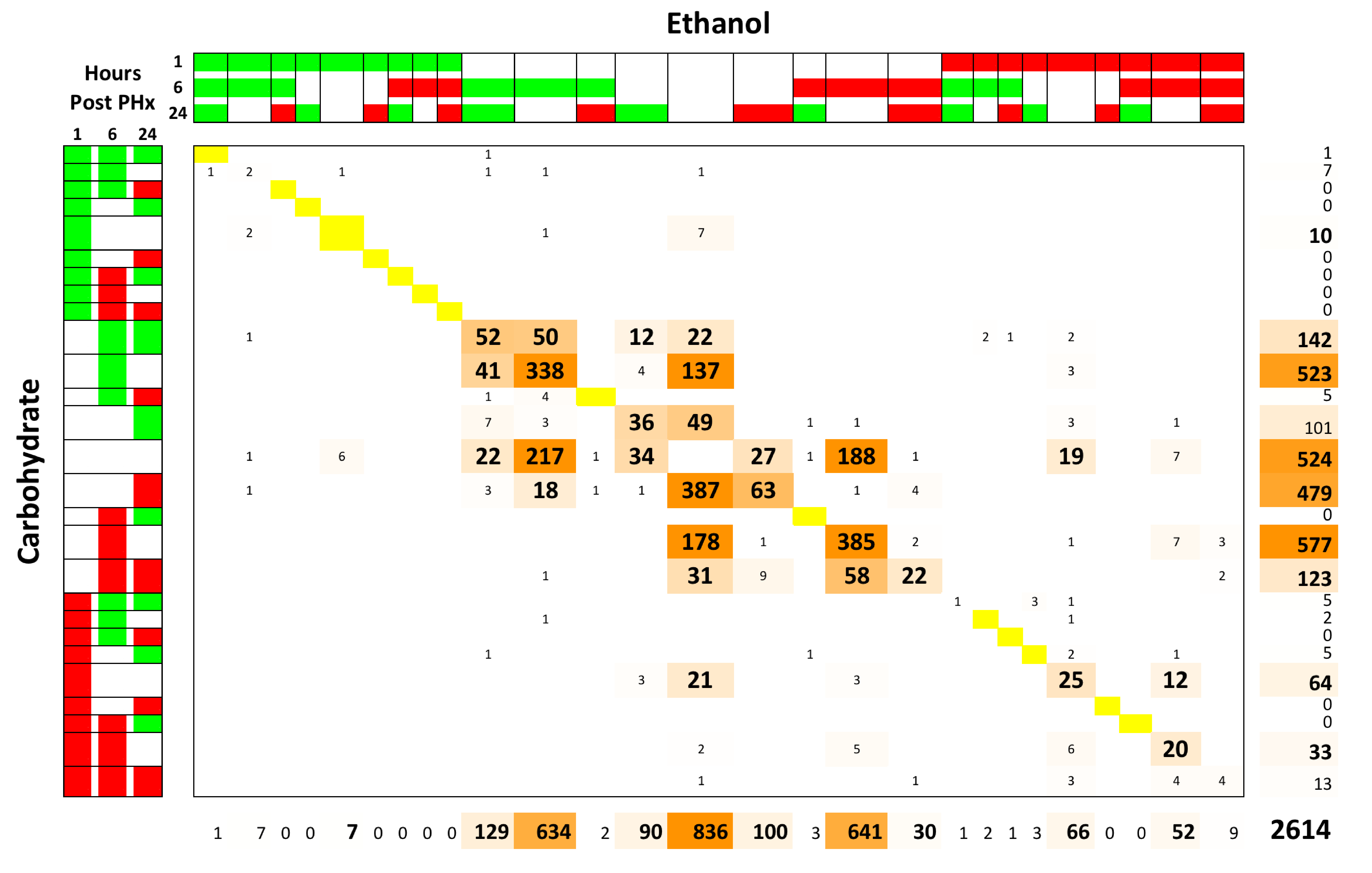

Supplement: Additional file 4: Figure S3. — A 27 × 27 Comparative Pattern Count (COMPACT) matrix comparing the Ethanol and Carbohydrate groups for a fold change threshold of 2.0. (TIF 2443 kb) [file 12864_2016_2492_MOESM4_ESM.tif]

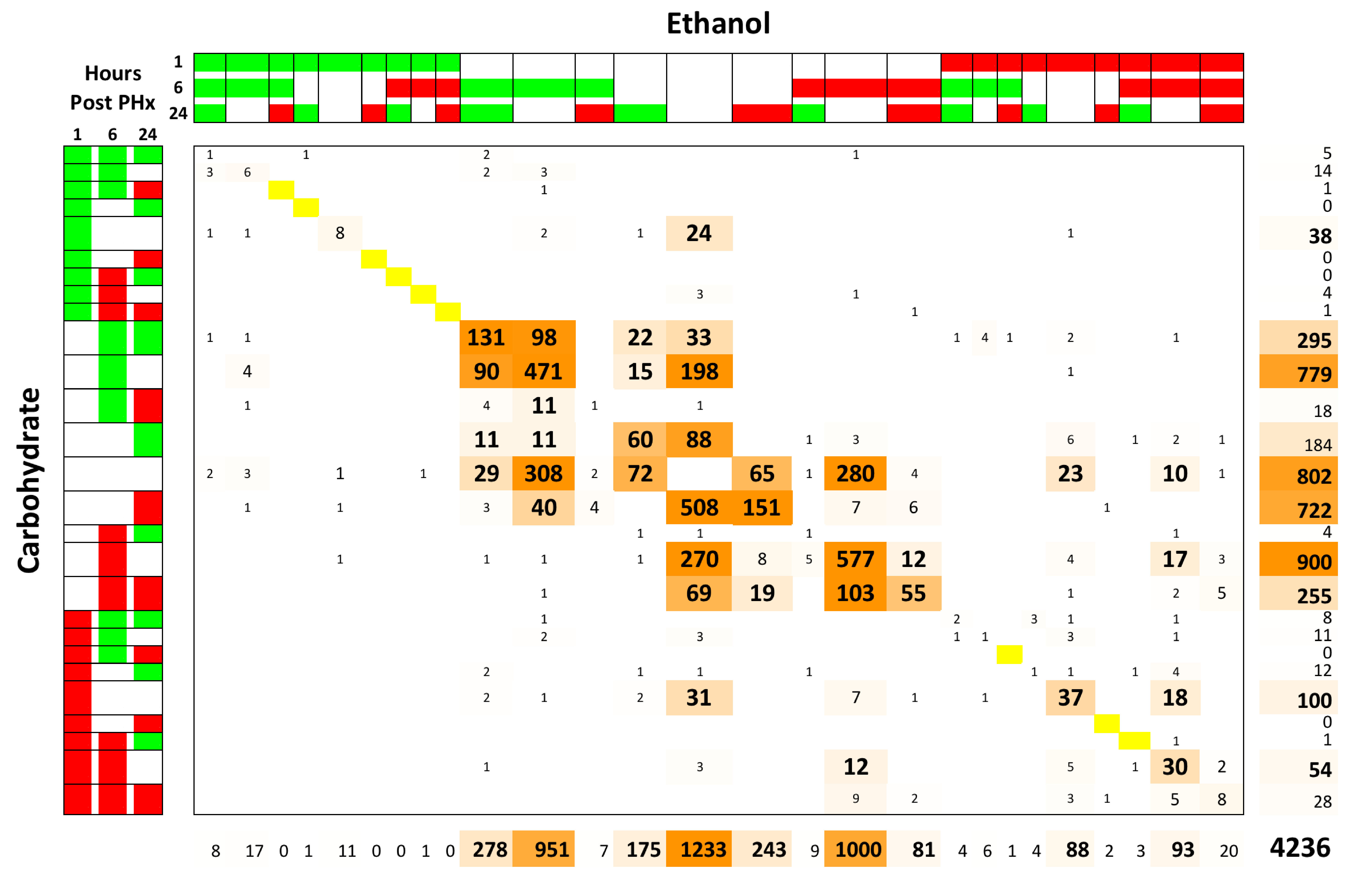

Supplement: Additional file 5: Figure S4. — A 27 × 27 Comparative Pattern Count (COMPACT) matrix comparing the Ethanol and Carbohydrate groups for a fold change threshold of 1.7. (TIF 2840 kb) [file 12864_2016_2492_MOESM5_ESM.tif]

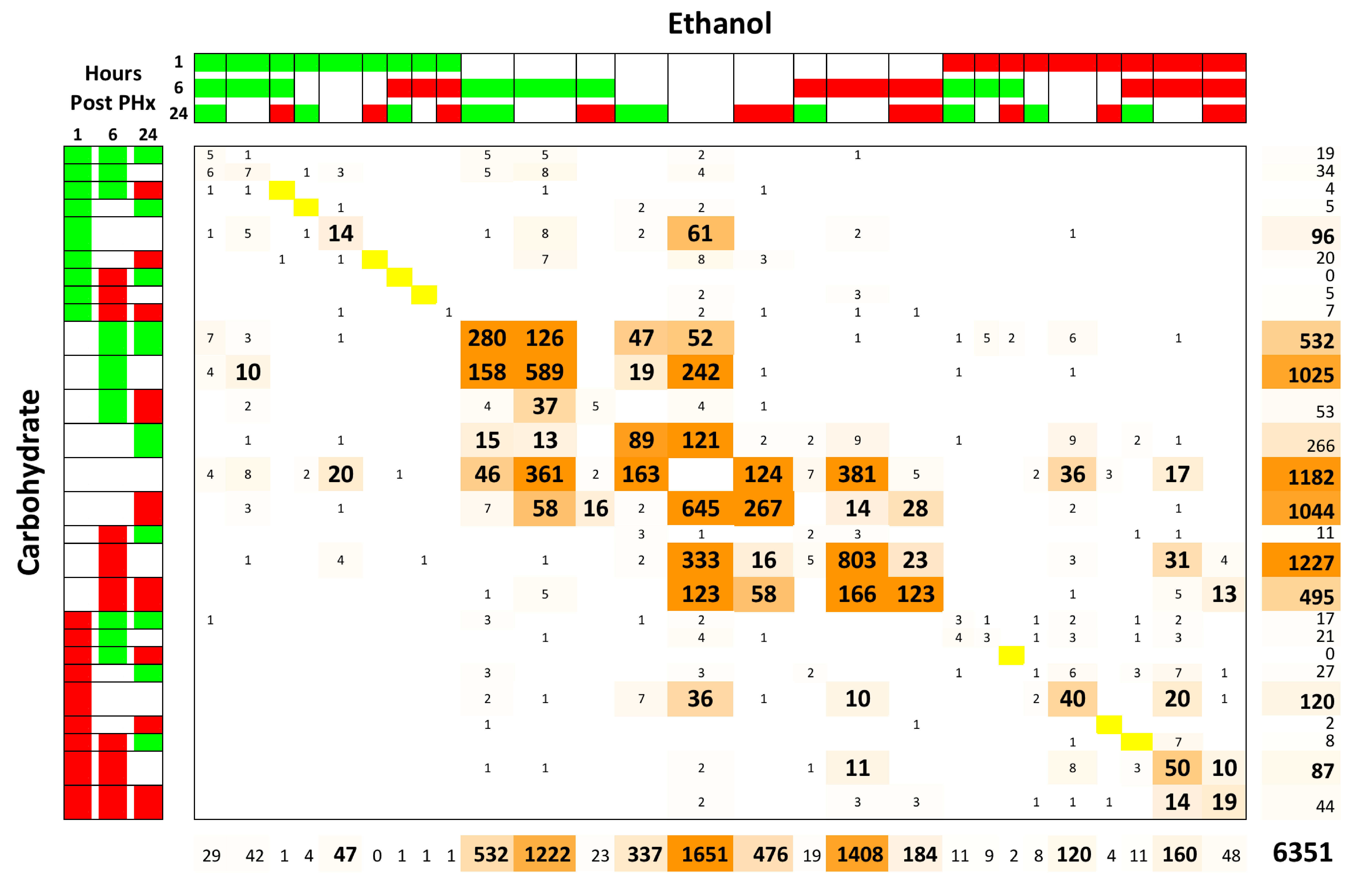

Supplement: Additional file 6: Figure S5. — A 27 × 27 Comparative Pattern Count (COMPACT) matrix comparing the Ethanol and Carbohydrate groups for a fold change threshold of 1.5. (TIF 3263 kb) [file 12864_2016_2492_MOESM6_ESM.tif]

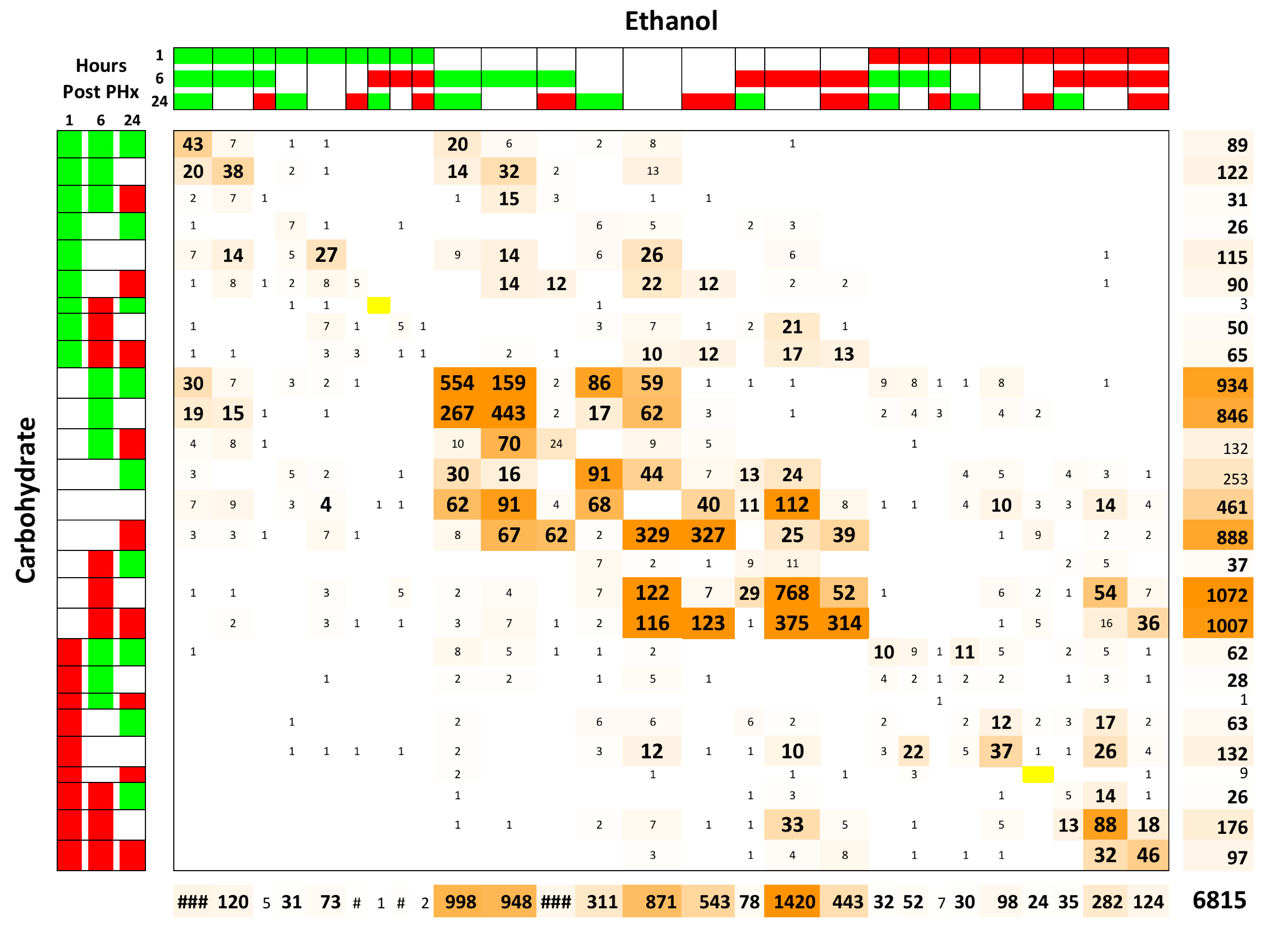

Supplement: Additional file 7: Figure S6. — A 27 × 27 Comparative Pattern Count (COMPACT) matrix comparing the Ethanol and Carbohydrate groups for a fold change threshold of 1.3. (TIF 4337 kb) [file 12864_2016_2492_MOESM7_ESM.tif]

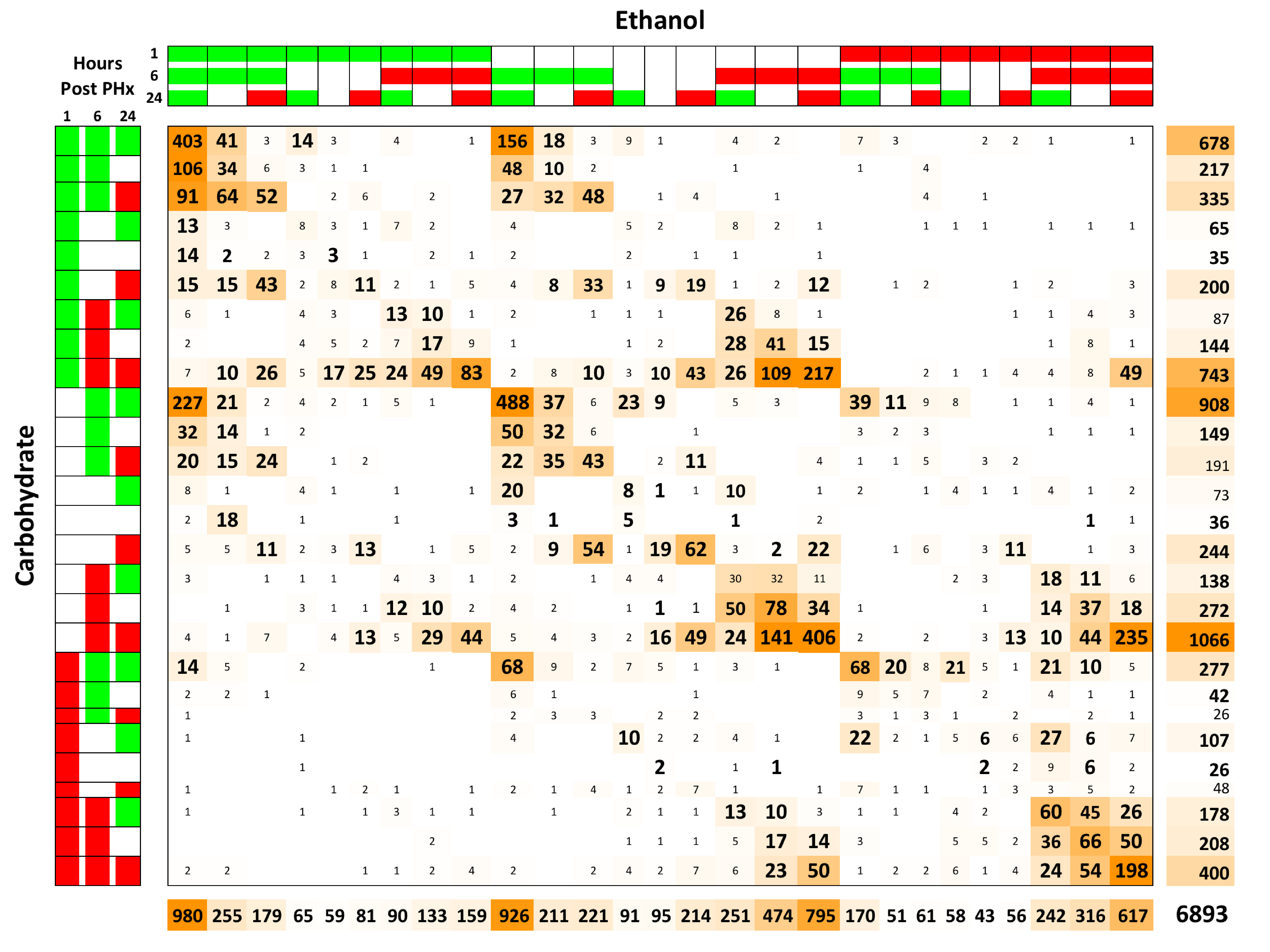

Supplement: Additional file 8: Figure S7. — A 27 × 27 Comparative Pattern Count (COMPACT) matrix comparing the Ethanol and Carbohydrate groups for a fold change threshold of 1.1. (TIF 5210 kb) [file 12864_2016_2492_MOESM8_ESM.tif]

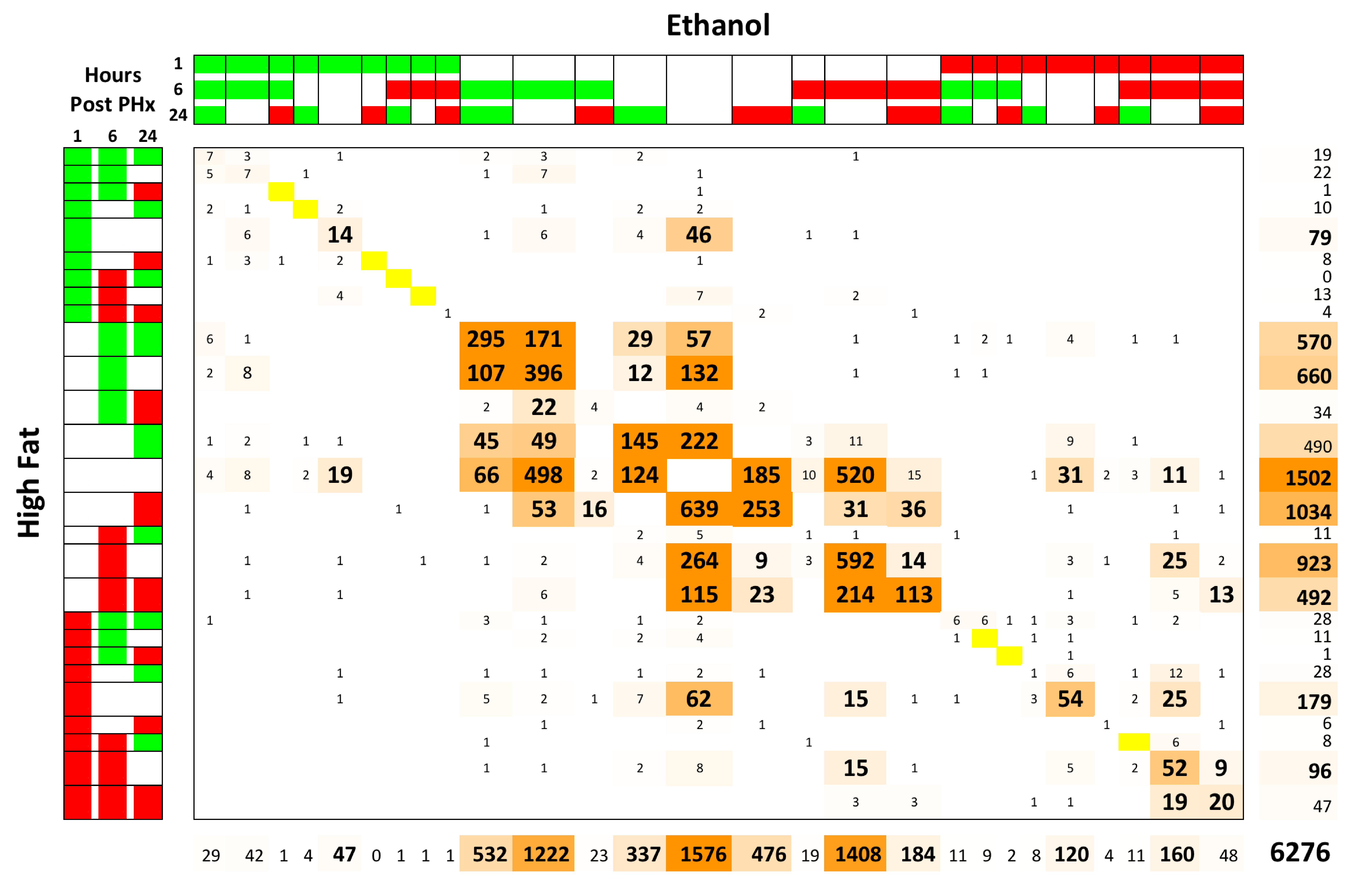

Supplement: Additional file 9: Figure S8. — A 27 × 27 Comparative Pattern Count (COMPACT) matrix comparing the Ethanol and High Fat groups for a fold change threshold of 1.5. (TIF 3185 kb) [file 12864_2016_2492_MOESM9_ESM.tif]

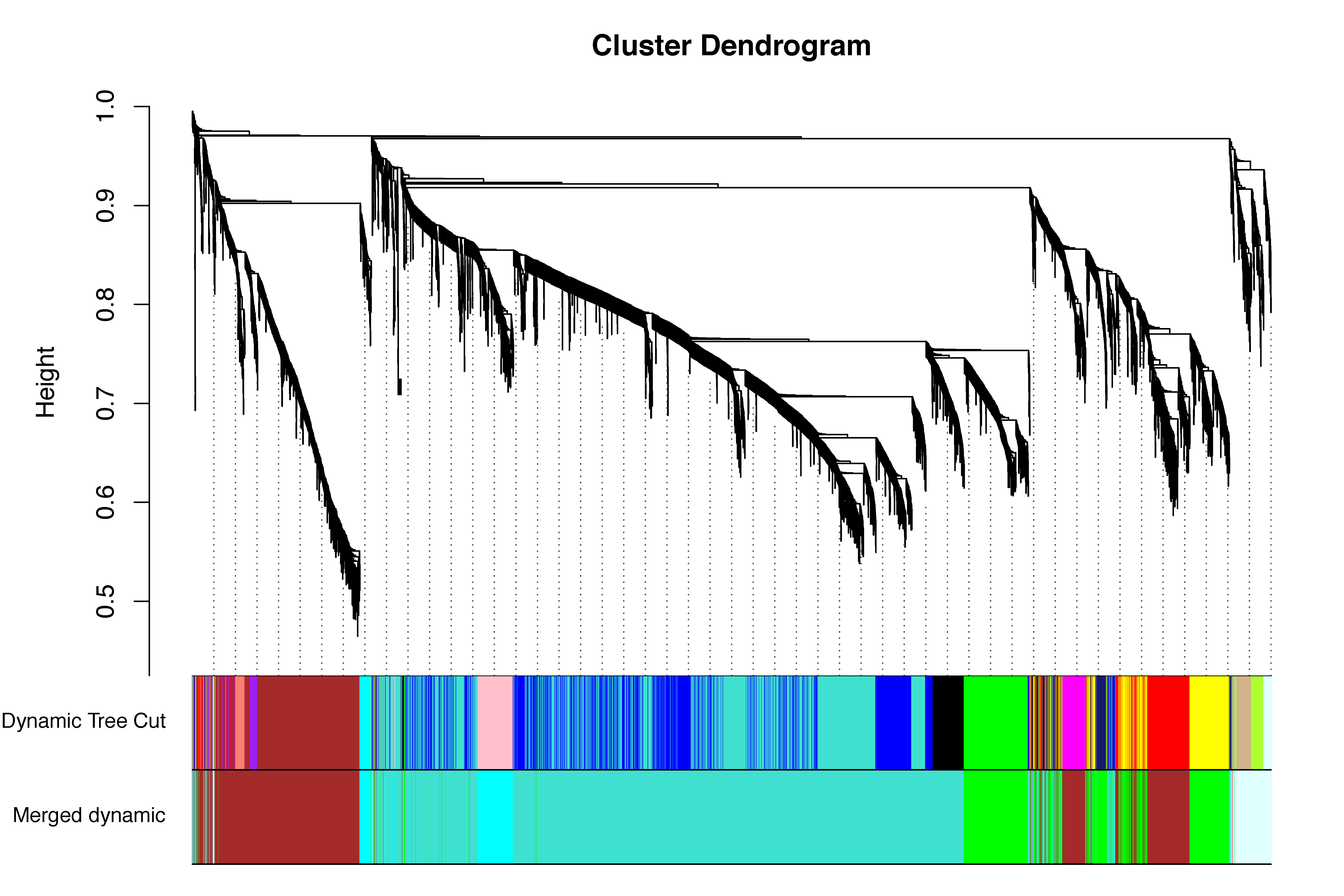

Supplement: Additional file 10: Figure S9. — Analysis of the gene expression data using WGCNA. Gene dendrogram obtained by average linkage hierarchical clustering. Module assignments using the Dynamic Tree Cut as well as the module merging process are shown underneath the dendrogram. (TIF 1841 kb) [file 12864_2016_2492_MOESM10_ESM.tif]
